# Discovery and Derivatization of Tridecaptin Antibiotics with Altered Host Specificity and Enhanced Bioactivity

**DOI:** 10.1021/acschembio.4c00034

**Published:** 2024-04-11

**Authors:** Nataliia V. Machushynets, Karol Al Ayed, Barbara R. Terlouw, Chao Du, Ned P. Buijs, Joost Willemse, Somayah S. Elsayed, Julian Schill, Vincent Trebosc, Michel Pieren, Francesca M. Alexander, Stephen A. Cochrane, Mark R. Liles, Marnix H. Medema, Nathaniel I. Martin, Gilles P. van Wezel

**Affiliations:** † Molecular Biotechnology, Institute of Biology, Leiden University, Leiden 2333 BE, The Netherlands; ‡ Biological Chemistry Group, Institute of Biology, Leiden University, Leiden 2333 BE, The Netherlands; § Bioinformatics Group, Wageningen University, Wageningen 6700 PB, The Netherlands; ∥ BioVersys AG, c/o Technologiepark, Basel CH-4057, Switzerland; # School of Chemistry and Chemical Engineering, Queen’s University of Belfast, Belfast BT9 5AG, United Kingdom; ⊥ Department of Biological Sciences, Auburn University, Auburn, Alabama 36849, United States; ∇ Department of Microbial Ecology, Netherlands Institute of Ecology, Wageningen 6700 PB, The Netherlands

## Abstract

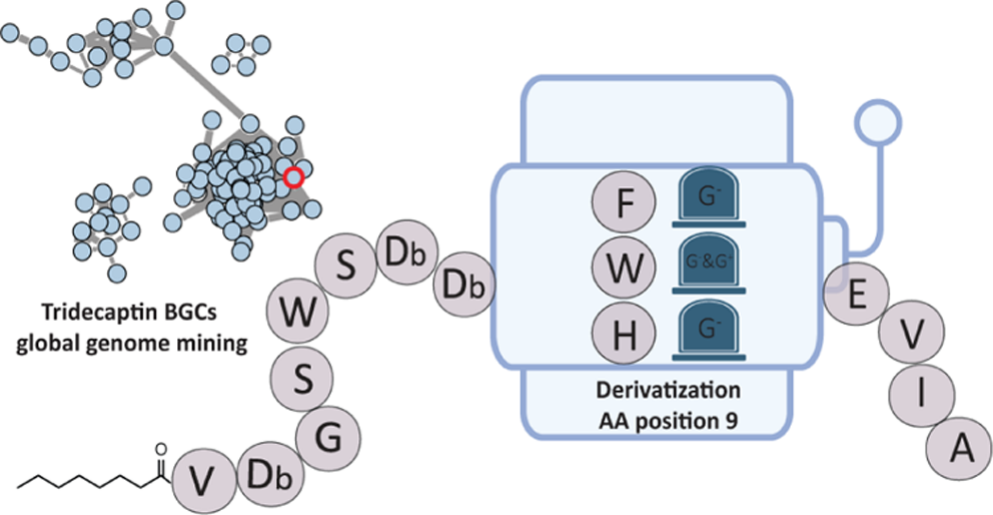

The prevalence of multidrug-resistant (MDR) pathogens combined with a decline in antibiotic discovery presents a major challenge for health care. To refill the discovery pipeline, we need to find new ways to uncover new chemical entities. Here, we report the global genome mining-guided discovery of new lipopeptide antibiotics tridecaptin A_5_ and tridecaptin D, which exhibit unusual bioactivities within their class. The change in the antibacterial spectrum of Oct-TriA_5_ was explained solely by a Phe to Trp substitution as compared to Oct-TriA_1_, while Oct-TriD contained 6 substitutions. Metabolomic analysis of producer *Paenibacillus* sp. JJ-21 validated the predicted amino acid sequence of tridecaptin A_5_. Screening of tridecaptin analogues substituted at position 9 identified Oct-His9 as a potent congener with exceptional efficacy against *Pseudomonas aeruginosa* and reduced hemolytic and cytotoxic properties. Our work highlights the promise of tridecaptin analogues to combat MDR pathogens.

## Introduction

The overuse of antibiotics over the past many decades has contributed to the rapid emergence and spread of antimicrobial resistance. Combined with the decline in the number of new clinically approved antibacterial drugs, infections caused by resistant bacteria are frequently difficult to treat.^1^ The ever-increasing prevalence of multidrug-resistant (MDR) bacteria is recognized by the World Health Organization (WHO) as an imminent threat to human health, particularly the Gram-negative critical priority pathogens *Acinetobacter baumannii*, *Pseudomonas aeruginosa*, and *Enterobacteriaceae*.^2^ Thus, there is an urgent need for novel antimicrobial agents, and the challenge lies in finding the undiscovered germs that may form the basis for our future medicines.^3,4^ Traditional high-throughput screening is becoming less attractive due to the issue of dereplication, rediscovering compounds that had already been identified before.^5,6^ However, next-generation sequencing has revealed that microbial genomes still harbor a huge underexplored biosynthetic potential of bacteria.^7,8^

Polymyxins are a very promising class of antibiotics that are used as a last line of therapy against MDR pathogens^9^ and are primarily produced by *Paenibacillus* species. With resistance against polymyxins inevitably increasing, we urgently need to search for potent alternatives. *Bacillus* and *Paenibacillus* produce a range of other classes of lipopeptides that act on MDR Gram-negative pathogens, including brevicidines, laterocidines, relacidines, paenibacterins, and tridecaptins.^10−14^ Of these, tridecaptins stand out as they possess a mechanism of action that is distinct from that of the aforementioned lipopeptides. In addition to interacting with lipopolysaccharides (LPS), tridecaptins also bind to Gram-negative lipid II and in doing so cause the disruption of the proton motive force.^15−18^ This unique dual mechanism of action reduces the likelihood of cross-resistance.^19^ Since the discovery of tridecaptin A in 1978, multiple tridecaptin variants with modifications at either the N-terminal fatty acid moiety or in the amino acid sequence have been reported. Tridecaptin A,^20^ tridecaptin B,^15^ tridecaptin C,^21^ and tridecaptin M^22^ are predominantly active against Gram-negative bacteria, while the recently discovered tridecaptin G^23^ has a broad-spectrum activity. Studies on natural tridecaptins include research on their biosynthesis, structure–activity relationships, their mechanism of action, and their synergy with other antibiotics.^14^ Structure–activity studies formed the basis for drug development studies to enhance their potency,^24,25^ expand their bioactivity spectrum,^26^ or increase their stability.^27^

In this regard, approaches that combine genome mining with variation in culturing conditions have proven to be a valuable way to achieve differential synthesis of NPs, followed by the metabolic profiling-based identification of the bioactivity of interest.^28^ However, a major challenge is to find the appropriate chemical triggers or ecological cues to elicit the production of cryptic antibiotics.^29−32^ An alternative way to investigate the potential of novel scaffolds predicted by genome mining is via organic synthesis or chemoenzymatic total synthesis.^26,33,34^ Genome mining tools such as antiSMASH^35^ allow interrogation of microbial genomes for the presence of biosynthetic gene clusters (BGCs) that specify the biosynthesis of natural products and subsequently predict the types of compounds that are derived from them, such as polyketides,^36^ ribosomally synthesized and post-translationally modified peptides (RiPPs),^37^ terpenes,^38^ or nonribosomal peptides (NRPs).^39^ In the case of nonribosomal peptide synthetases (NRPSs), NRPS modules and the amino acids predicted to be incorporated by the A-domain in each module can be predicted using NRPS-specific prediction algorithms, such as NRPSpredictor2^40^ or SANDPUMA^41^ or machine learning techniques trained on a set of A-domains with known specificities.^42^ With the power of modern synthetic organic chemistry and the increasing accuracy of natural product structure prediction algorithms, it is increasingly possible to chemically synthesize new bioactive molecules based on BGC sequences.^33^

Here, we report a global genome-mining approach that led to the discovery of novel tridecaptins. Bioinformatics analysis of 785 bacterial genomes identified novel tridecaptins, tridecaptin A_5_ and tridecaptin D. Unexpectedly, 2 synthetic analogues Oct-TriD and Oct-TriA_5_ efficiently killed both Gram-negative and Gram-positive bacteria. The increased bioactivity spectrum of tridecaptin Oct-TriA_5_ compared to that of Oct-TriA_1_ also correlated with hemolytic and cytotoxic activity and could be attributed to a single amino acid substitution at position 9. In contrast to Oct-TriA_1_, the broadened antimicrobial spectrum of Oct-TriA_5_ and Oct-TriD is caused by its increased membrane disruptive capacity against Gram-positive pathogens. Subsequent screening of other amino acids at position 9 led to the discovery of the potent tridecaptin analogue Oct-TriHis9 with reduced hemolytic and cytotoxic properties and potent activity against *P. aeruginosa*.

## Results and Discussion

### Large-Scale Network Analysis and Bioinformatic Prediction of Novel Tridecaptin BGCs

**Figure 1:**
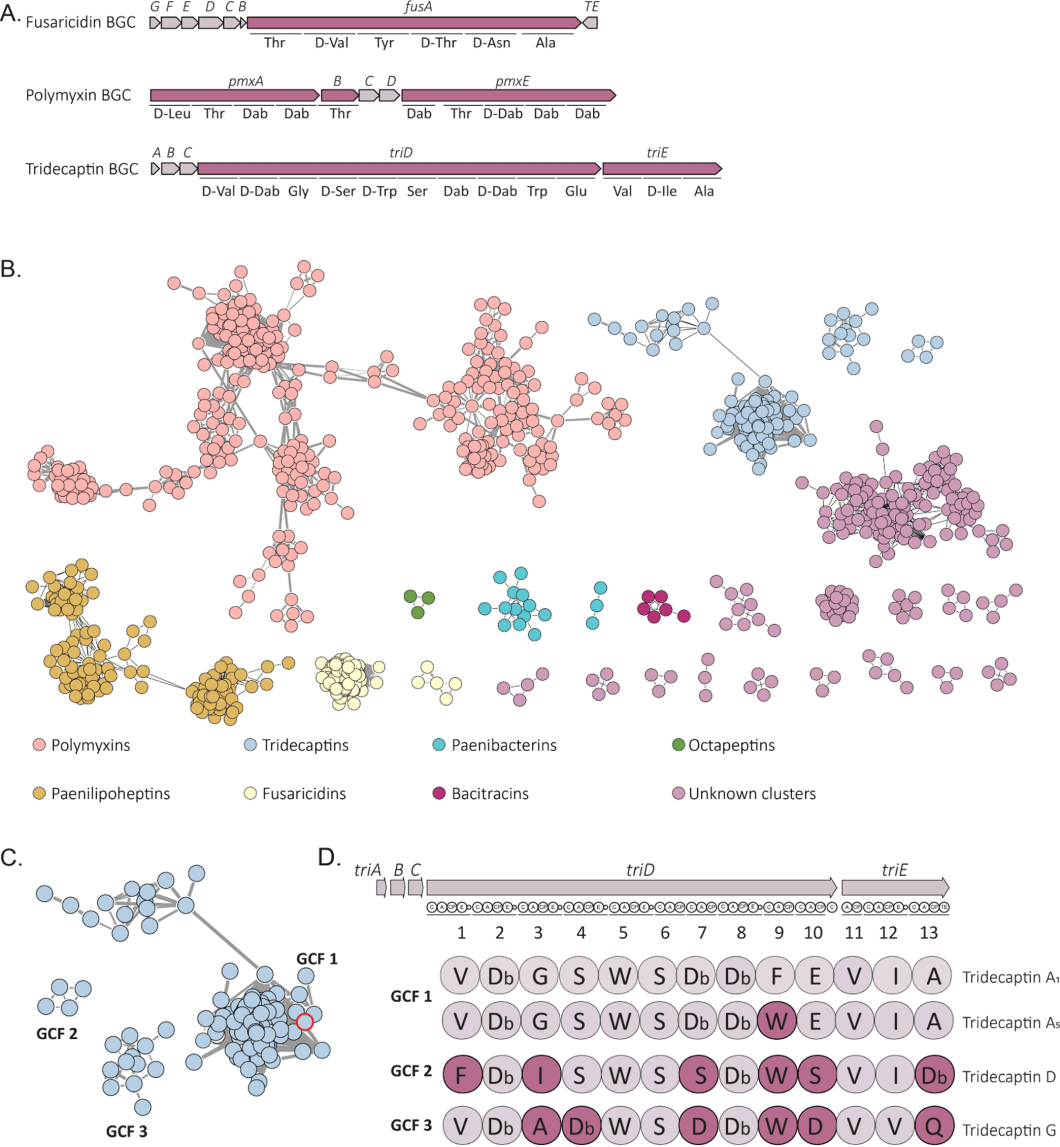
A. Organization of the NRPS BGCs predicted in *Paenibacillus* sp. JJ-21 genome that encodes the biosynthesis of lipopeptides. B. BiG-SCAPE sequence similarity network (SSN) (c 0.25) containing validated NRPS BGCs of *Paenibacillus* spp. visualized in Cytoscape. Each node represents one NRPS BGC identified by antiSMASH. Singletons and single links are not shown. C. Enlarged gene cluster families (GCFs) of tridecaptin BGCs. Red circles indicate the tridecaptin BGC detected in the genome of *Paenibacillus* sp. JJ-21. D. Scheme of the tridecaptin BGC and amino acid sequences of tridecaptin A_1_, new tridecaptin A_5_, tridecaptin D, and tridecaptin G. The sequences of their peptide moieties were predicted from the active site sequences of their A domains. Note the peptide sequences of tridecaptin A_5_ and the well-known tridecaptin A_1_ differ exclusively in one amino acid at position 9. Db: 2,4-diaminobutyric acid.

*Paenibacillus* sp. JJ-21 is a gifted natural product producer isolated from the corn rhizosphere and identified as an antibacterial metabolite producer based on its antagonistic potential against polymyxin-resistant *Escherichia coli* ATCC 25922 harboring the plasmid-mediated *mcr-1* resistance gene. The *Paenibacillus* sp. JJ-21 genome was sequenced using the PacBio platform. Assembly of the PacBio reads with Falcon (version 1.8.1)^43^ resulted in a single contig of 6.2 Mb (GenBank accession number: CP132974). The *Paenibacillus* sp. JJ-21 genome was analyzed using antiSMASH 6.0.1^44^ to obtain an overview of the predicted BGCs. AntiSMASH predicted a total of 18 BGCs, including 3 for NRPSs (Figure 1A). These NRPS BGCs were predicted to encode the biosynthesis of fusaricidin, tridecaptin, and polymyxin , respectively, based on their high similarity (identical gene order and >75% nucleotide sequence identity) to characterized clusters (listed in MIBiG).^45^ An *in silico* analysis of the A domain substrate specificity of these BGCs was conducted with software tool PARAS (v0.0.4, available at https://github.com/BTheDragonMaster/paras) to predict the amino acid composition of the peptide scaffolds (Table S1). Surprisingly, the amino acid sequence predicted from the tridecaptin BGC differed from that of the well-known tridecaptin A_1_ and contained Trp instead of Phe in position 9 (Table S2).

To gain insight into the chemical space of NRPSs and specifically tridecaptin BGCs, we bioinformatically analyzed 785 complete genomes from *Paenibacillus* spp. for BGCs encoding NRPSs. The genome of *Paenibacillus* sp. JJ-21 was included as the reference genome in the data set. To visualize the diversity, distribution, and NRPS novelty, a BiG-SCAPE^46^ sequence similarity network (SSN) was then constructed, which consisted of 4,367 NRPS BGCs forming 1609 gene cluster families (GCFs) (Figure 1B).

Genetic variation of BGCs within GCFs is often directly associated with structural differences between their molecular products, and even small chemical variations can lead to different biological activities.^47^ We also visualized the modular architecture of NRPS assembly lines, revealing the order and number of modules for each NRPS synthase that in turn helped to determine the class of molecules they encode. The BiG-SCAPE sequence similarity network highlighted various known classes of NRPSs, such as polymyxins (3 NRPSs, 10 modules), tridecaptins (2 NRPSs, 13 modules), paenilipoheptins (3 NRPSs, 7 modules), fusaricidins (1 NRPS, 6 modules), paenibacterins (4 NRPSs, 13 modules), octapeptins (3 NRPS, 8 modules), bacitracins (3 NRPS, 12 modules), and cilagicins (3 NRPS, 12 modules), but 20 percent of the detected BGCs could not be linked to known compounds.^45^

Surprisingly, 3 GCFs contained BGCs with 13 NRPS modules and were predicted as tridecaptin BGCs based on the antiSMASH and BiG-SCAPE results. The assembly line of tridecaptins is subdivided into 2 peptide synthetases, namely, TriD and TriE, consisting of 10 and 3 modules, respectively.^48^ Notably, substrate specificity predictions by PARAS-indicated that the tridecaptin BGCs within GCF 1 encoded not only the previously characterized tridecaptin A but also a new analogue, designated tridecaptin A_5_, which should incorporate Trp in position 9, and was detected in the genome of *Paenibacillus* sp. JJ-21 (Figure 1D, Table S3). Additionally, we discovered another new structurally distinct analogue of tridecaptins, which we designated as tridecaptin D and is predicted to be encoded by the tridecaptin BGCs of GCF 2. GCF 3 represented BGCs corresponding to the recently published tridecaptin G.^23^ Tridecaptin D differs from tridecaptin A_1_ at 6 amino acid positions, with 5 positions of the molecule featuring amino acids that are unique among all known tridecaptins identified so far, namely, Phe1, Ile3, Ser7, Ser10, and Dab13. We used antiSMASH to predict which modules in the BGC for tridecaptin D contained epimerization domains, i.e., domains that catalyze the conversion from - to -amino acids. Modules 1–5 and 8 of TriD and module 12 of TriE, which incorporate Phe, Dab, Ile, Ser, Trp, Dab, and Ile, respectively, were all predicted to contain epimerization domains (Table S3).

### Identification of Tridecaptin A_5_*In Vivo* Using Metabolomics

To validate the bioinformatic prediction of the amino acid sequence of tridecaptin A_5_, the lipopeptide was detected in the cultures of *Paenibacillus* sp. JJ-21 and analyzed by LC-MS/MS. For this, *Paenibacillus* sp. JJ-21 was fermented in liquid TSB media, and biomass was collected by centrifugation and subsequently extracted with acidified 70% IPA. LC-MS/MS analysis identified fusaricidins and polymyxins as the major secondary metabolites. Considering that all known tridecaptins contain several positively charged diaminobutyric acids (Dab), we searched for the MS/MS spectra that contained a *b* ion with *m*/*z* 101.0709. Besides polymyxins that also contained multiple Dab residues, we detected a compound with *m*/*z* 539.9712, corresponding to an [M + 3H]^3+^ ion. Based on the biosynthetic features derived from the genome sequence of *Paenibacillus* sp. JJ-21, we initiated *de novo* sequencing of the compound with the *m*/*z* 539.9712 from the y and b ion series (Figure S1). The MS/MS spectrum showed a low-mass ion region containing a Trp immonium ion with an *m*/*z* 159.0912 as well as internal fragment ions. Fragment analysis of the *y* and *b* ions yielded an amino acid sequence that matched that predicted for tridecaptin A_5_, which is FA-d-Val–d-Dab–Gly–d-Ser–d-Trp–l-Ser–l-Dab–d-Dab–l-Trp–l-Glu–l-Val–d-Ile–l-Ala. The MS/MS spectrum revealed the presence of a hydroxy-containing C11 fatty acid in tridecaptin A_5_, similar to the previously reported 3-hydroxy-methyldecanoic fatty acids found in tridecaptin A_3_ and tridecaptin A_4_.^48^ The MS/MS studies did not allow us to discriminate between Leu, Ile, and *allo*-Ile in position 12 of the tridecaptin sequence. Here, the assignment was performed according to the structure prediction from our genome mining studies and comparison with the literature data.^48^ Additionally, the MS results do not allow optical isomers to be distinguished. Therefore, the configurations of the residues of tridecaptin A_5_ were predicted from the domain organization of the modules along the assembly lines.

### Chemical Synthesis and Antimicrobial Testing of Tridecaptin Analogues

**Figure 2:**
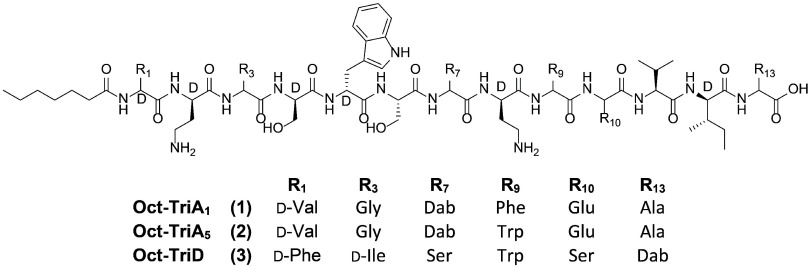
Structures of synthetic tridecaptin A variants Oct-TriA_1_ (**1**), Oct-TriA_5_ (**2**), and Oct-TriD (**3**).

Because of the low levels of production of tridecaptin A_5_ by *Paenibacillus* sp. JJ-21, we decided to produce synthetic analogues of the peptides by solid-phase peptide synthesis (SPPS). The added value of this approach is that it also allows the synthesis and direct comparison of analogues, chosen based on the substrate specificity and stereochemical predictions generated from the analysis of the tridecaptin BGCs. Previously, it was demonstrated that a synthetic analogue of tridecaptin A_1_ (TriA_1_) wherein the N-terminal acyl moiety was replaced with octanoic acid retained the full activity of the natural product against ESKAPE pathogens (*Enterococcus faecium*, *Staphylococcus aureus*, *Klebsiella pneumoniae*, *A. baumannii*, *P. aeruginosa*, and *Enterobacter* species).^25^ For this reason, in the present studies, the chiral lipid tail of the tridecaptins was also replaced with the octanoyl chain, which is more accessible than natural lipids and enables larger quantities of peptide to be obtained for biological studies. Linear synthetic analogues of tridecaptin A_1_, tridecaptin A_5_, and tridecaptin D were synthesized and designated as Oct-TriA_1_ (**1**), Oct-TriA_5_ (**2**), and Oct-TriD (**3**), respectively (Table S4, Figures 2 and S2–S4).

Subsequently, Oct-TriA_1_, Oct-TriA_5_, and Oct-TriD were assayed for activity against the ESKAPE pathogens. MIC values were determined using broth-dilution assays (Table 1). Oct-TriA_1_ displayed potent activity against most of the Gram-negative test strains but was less active against Gram-positive bacteria, similar to known tridecaptins (i.e., tridecaptins A, B, C, and M).^25^ Surprisingly, Oct-TriD showed significant bioactivity against the Gram-positive species *Staphylococcus aureus*, *Enterococcus faecalis* and *Enterococcus faecium*, with an MIC between 4–8 μg/mL, in addition to moderate activity against Gram-negative pathogens. To the best of our knowledge, Oct-TriD is the first tridecaptin analogue that is predominantly active against Gram-positive bacteria. Moreover, Oct-TriA_5_ showed broad-spectrum bioactivity, similar to the recently published synthetic analogue syn-CNRL5 and natural tridecaptin G.^23,26^ Specifically, Oct-TriA_5_ exhibited activity against Gram-negative ESKAPE pathogens, with MICs ranging from 2 to 16 μg/mL. Notably, a single amino acid substitution at position 9 from Phe in Oct-TriA_1_ to Trp in Oct-TriA_5_ changed the bioactivity profile of Oct-TriA_5_ from being limited to Gram-negative bacteria to broad-spectrum activity.

**Table 1:** *In Vitro* Minimum Inhibitory Concentrations (MICs) of Oct-TriA_1_, Oct-TriA_5_, and Oct-TriD^a^

strain			MIC			
		species	Oct-TriA_1_ (1)	Oct-TriA_5_ (2)	Oct-TriD (3)	colistin
BV18	UAMS-1625	*S. aureus*	32	8	16	>64
BV249	ATCC 29213	*S. aureus*	32	8	8	>64
BV1402	1126387	*S. aureus*	32	8	16	>64
BV29	ATCC 29212	*E. faecalis*	32	8	8	>64
BV99	ATCC 25922	*E. faecalis*	32	8	8	>64
BV144	IHMA 890472	*E. faecium*	16	8	4	>64
BV159	ATCC 51559	*E. faecium*	32	8	4	>64
BV94	IHMA 777621	*A. baumannii*	8	4	8	64
BV160	ATCC 17978	*A. baumannii*	8	4	16	1
BV374	HUMC1	*A. baumannii*	16	8	16	1
BV23	ATCC 25922	*E. coli*	2	2	32	1
BV1475	1220120	*E. coli*	2	4	16	0.5
BV1469	1214245	*E. coli*	2	4	16	32
BV306	ATCC 13883	*K. pneumoniae*	4	4	8	1
BV1445	1227947	*K. pneumoniae*	4	4	>64	0.5
BV1447	1228586	*K. pneumoniae*	4	4	64	64
BV34	PAO1	*P. aeruginosa*	64	16	>64	2
BV1551	1226072	*P. aeruginosa*	8	8	32	0.5
BV1544	1218019	*P. aeruginosa*	4	4	32	>64
Hemolysis EC50 [μg/mL]			102.2	6.9	2.2	n/a
IC50 on HepG2 w/o FCS [μg/mL]			109.6	11.4	6.2	n/a

a MIC values reported in units of μg/mL.

In addition, tridecaptins were tested for their hemolytic and cytotoxic activities (Table 1). Oct-TriA_5_ and Oct-TriD showed increased hemolytic and cytotoxic activity compared to Oct-TriA_1_, suggesting that the altered spectrum of activity of Oct-TriA_5_ and Oct-TriD is due to unspecific membrane lysis properties and emphasizing the importance of the amino acid in position 9 for selectivity toward antibacterial activity.

### Mode of Action on Gram-Positive and Gram-Negative Bacteria

Tridecaptin A_1_ exerts its bactericidal effect on Gram-negative bacteria by binding to lipid II on the surface of the inner membrane and by disrupting the proton motive force.^18^ Tridecaptin A_1_ binds to both Gram-positive and Gram-negative lipid II although it has a much higher affinity for the Gram-negative analogue.^25,49^ To assess if Oct-TriA_5_ analogues bind to lipid II, we performed *in vitro* lipid II antagonization assays. Gram-positive lipid II, containing lysine at position 3 of the pentapeptide, was prepared by the total chemical synthesis. The bioactivity of Oct-TriA_5_ against *S. aureus* USA300 (MRSA) was evident with an MIC of 8 μg/mL. However, the addition of lipid II significantly reduced the efficacy of Oct-TriA_5_, as the growth of *S. aureus* USA300 (MRSA) was not inhibited at the concentration of 8x MIC. (Figure S5). This indicates that Oct-TriA_5_ binds to lipid II of Gram-positive bacteria.

**Figure 3:**
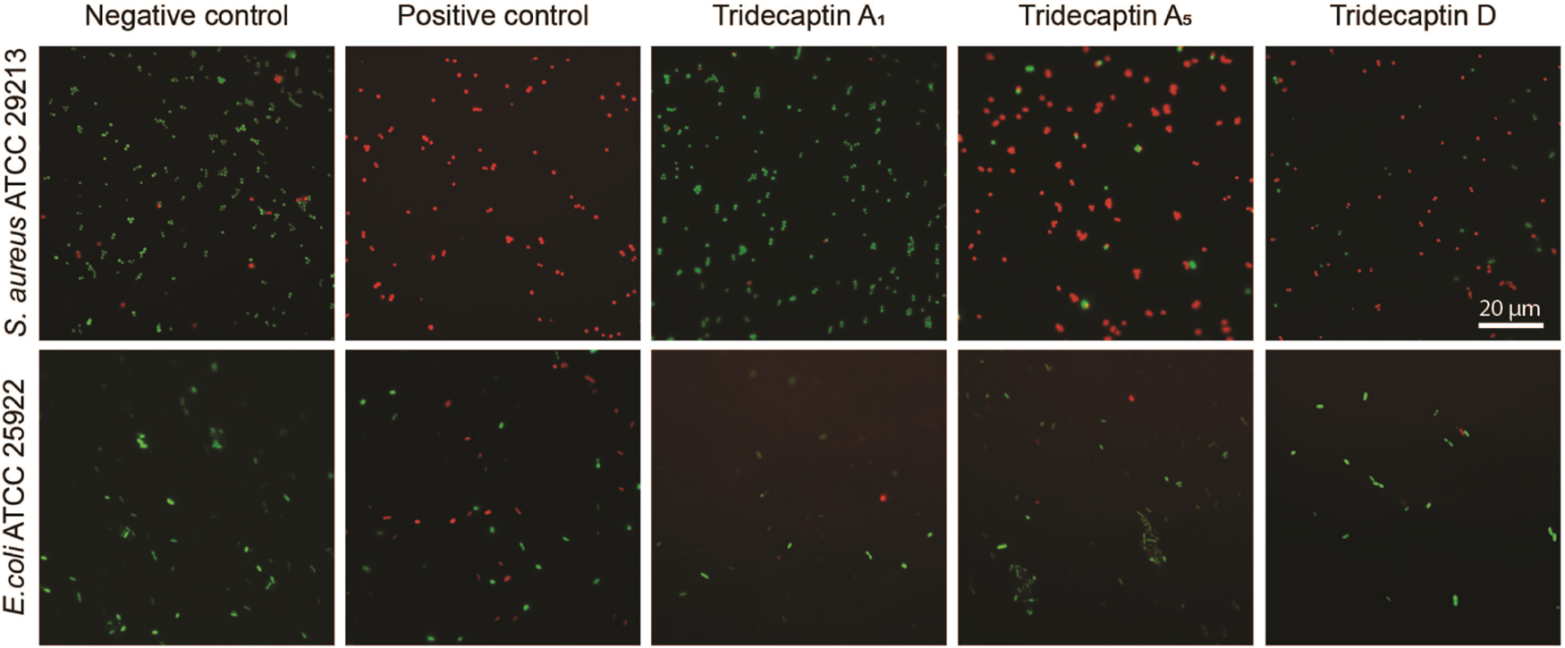
LIVE/DEAD staining of *S. aureus* ATCC 29213 and *E. coli* ATCC 25922 cells in the control conditions and after being exposed to nisin/polymyxin B or tridecaptin analogues Oct-TriA_1_, Oct-TriA_5_, and Oct-TriD. Green color indicates the cells with intact membranes, while cells with compromised membranes are stained in red. Note that after the treatment with Oct-TriA_5_ and Oct-TriD *S. aureus* cells were stained red indicating the membrane disruption.

To monitor the effect of tridecaptins on the membrane permeability of Gram-positive and Gram-negative bacteria, we performed a LIVE/DEAD staining assay with SYTO 9 and propidium iodide with *S. aureus* ATCC 29213 and *E. coli* ATCC 25922, as published previously.^50^ Microscopic analysis demonstrated that the vast majority of *S. aureus* cells when exposed to Oct-TriA_5_ and Oct-TriD exhibited red staining comparable to that of the positive control (Figure 3).

*S. aureus* cells incubated with Oct-TriA_5_ and Oct-TriD had lysed, indicative of nonspecific membrane disruption, while those incubated with Oct-TriA_1_ were still alive, suggesting that Oct-TriA_1_ did not have any influence on the membrane permeability. Treatment of *E. coli* cells with Oct-TriA_1_, Oct-TriA_5_, or Oct-TriD did affect the membrane integrity, while after the same treatment with polymyxin (4 μg/mL; 2 × MIC) resulted in the death of half of the *E. coli* cells. These data strongly suggest that Oct-TriA_5_ and Oct-TriD act on Gram-positive bacteria by disrupting the cellular membrane. This is most likely explained by the presence of Trp9 in Oct-TriA_5_ instead of Phe9 in Oct-TriA_1_, which made the Oct-TriA_5_ more hydrophobic and therefore increased its membrane disruption capacity.

### Amino Acid Substitutions in Tridecaptin A at Position 9 Impacting Bioactivity and Cytotoxicity

Considering the striking difference in the spectrum of activity between tridecaptin A_1_ (active against Gram-negatives) and A_5_ (active against both Gram-positives and Gram-negatives), we decided to look into the impact of changes in position 9 on the bioactivity. To do so, we synthesized a series of analogues wherein residue 9 was changed into Gly, Ala, Val, Ile, Ser, Tyr, Glu, His, or Dab and compared these new variants to Oct-TriA_1_ and Oct-TriA_5_ for bioactivity as well as their hemolytic and cytotoxic activity (Table S4, Figures S6–S14).

The synthetic analogues exhibited significant variation in their MIC values against the pathogens tested (Table 2). Hemolytic and cytotoxic activities also differed significantly, which provided further proof of concept that indeed the residue in position 9 plays a critical role in the hemolytic and cytotoxic activity of tridecaptin A (Table 2).

**Table 2:**
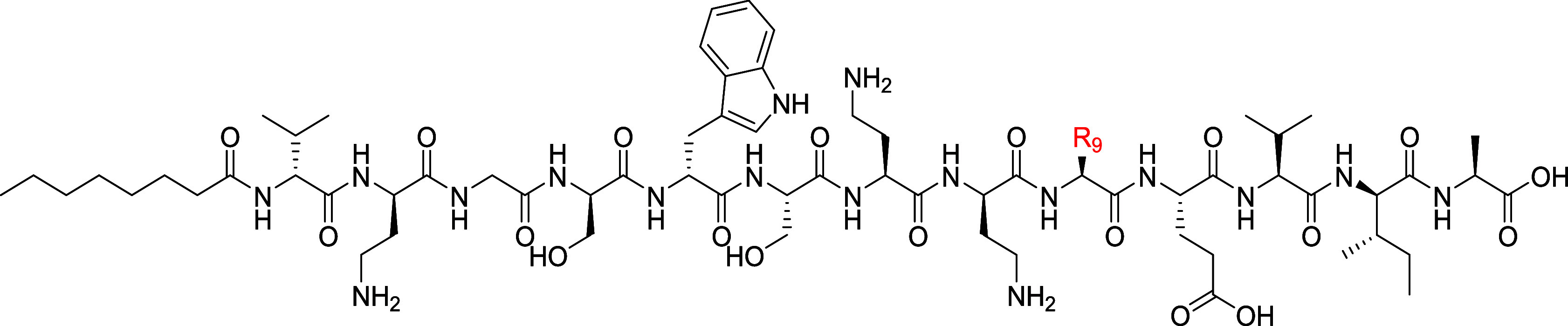
*In Vitro* Minimum Inhibitory Concentrations (MICs) of Synthetic Tridecaptin Analogues^a^

strain		MIC									
		Oct-Gly9 (4)	Oct-Ala9 (5)	Oct-Val9 (6)	Oct-Ile9 (7)	Oct-Ser9 (8)	Oct-Tyr9 (9)	Oct-Glu9 (10)	Oct-His9 (11)	Oct-Dab9 (12)	colistin
UAMS-1625	*S. aureus*	>64	>64	>64	>64	>64	>64	>64	>64	>64	>64
ATCC 29213	*S. aureus*	>64	>64	>64	64	>64	>64	>64	>64	>64	>64
1126387	*S. aureus*	>64	>64	>64	>64	>64	>64	>64	>64	>64	>64
ATCC 29212	*E. faecalis*	>64	>64	64	64	>64	64	>64	>64	>64	>64
ATCC 25922	*E. faecalis*	>64	>64	>64	64	>64	64	>64	>64	>64	>64
IHMA 890472	*E. faecium*	>64	>64	>64	32	>64	32	>64	>64	>64	>64
ATCC 51559	*E. faecium*	>64	>64	64	64	>64	64	>64	>64	>64	>64
IHMA 777621	*A. baumannii*	>64	>64	32	16	>64	32	>64	>64	>64	64
ATCC 17978	*A. baumannii*	>64	>64	32	16	>64	32	>64	>64	>64	1
HUMC1	*A. baumannii*	>64	>64	32	16	>64	32	>64	>64	>64	1
ATCC 25922	*E. coli*	16	16	4	2	32	2	>64	16	>64	1
1220120	*E. coli*	16	8	4	2	32	4	64	8	>64	0.5
1214245	*E. coli*	16	16	4	2	64	2	>64	16	>64	32
ATCC 13883	*K. pneumoniae*	16	8	2	2	64	4	>64	16	>64	1
1227947	*K. pneumoniae*	32	16	8	4	>64	4	>64	32	>64	0.5
1228586	*K. pneumoniae*	8	8	8	4	8	4	>64	8	>64	64
PAO1	*P. aeruginosa*	64	64	64	64	>64	64	>64	32	>64	2
1226072	*P. aeruginosa*	32	32	16	16	32	4	>64	4	8	0.5
1218019	*P. aeruginosa*	>64	>64	16	16	32	4	>64	2	>64	>64
Hemolysis EC50 [μg/mL]		>128	>128	>128	>128	>128	65.6	>128	>128	>128	n/a
IC50 on HepG2 w/o FCS [μg/mL]		>128	>128	>128	>110.2	>128	64	>128	>128	>128	>128

a MIC values reported in units of μg/mL.

Of particular interest was Oct-His9, which efficiently inhibited colistin-resistant *P. aeruginosa* 1218019, with an MIC of 2 μg/mL. Furthermore, Oct-His9 showed lower hemolytic and cytotoxic activity compared to Oct-TriA_1_. Conversely, synthetic analogues Oct-Gly9, Oct-Ala9, Oct-Ser9, Oct-Glu9, and Oct-Dab9 showed no antibacterial activity against Gram-positive bacteria at a concentration of 64 μg/mL and were moderately active against Gram-negative pathogens, with MICs ranging from 8 to 64 μg/mL. Similar to Oct-TriA_5_ and Oct-TriD, variants Oct-Tyr9 and Oct-Ile9 showed moderate bioactivity against Gram-positives and high cytotoxic and hemolytic activity. Synthetic analogues with Val and Ile at position 9 shared bioactivity, cytotoxic, and hemolytic profiles with Oct-TriA_1_. Taken together, our data highlight position 9 as a key residue for the spectrum and degree of bioactivity as well as the cytotoxicity of tridecaptin A.

## Concluding Remarks

We report here the discovery of new lipopeptide antibiotics tridecaptin A_5_ and tridecaptin D. Bioinformatics analysis of 785 complete *Paenibacillus* genomes revealed the BGC for tridecaptin A_5_ in the genome of *Paenibacillus* sp. JJ-21. Thorough examination of the adenylation domains encoded by tridecaptin A_5_ BGC allowed us to predict the amino acid sequence of the peptide scaffold; this was then matched to the natural product produced by *Paenibacillus* sp. JJ-21. Subsequent comparison of the tridecaptin A_5_ BGC with tridecaptin A_1_ revealed that they differed exclusively in module 9 of *triD*. Based on the PARAS substrate specificity predictions, we synthesized tridecaptin analogues Oct-TriA_5_, Oct-TriD, and reference Oct-TriA_1_ to evaluate their antibacterial activity against a panel of ESKAPE pathogens. Surprisingly, while tridecaptins are primarily active against Gram-negative bacteria, Oct-TriA_5_ possessed significant activity against both Gram-positive and Gram-negative bacteria, and Oct-TriD was primarily active against Gram-positive pathogens. Particularly striking is that a single amino acid substitution at position 9, from Phe in Oct-TriA_1_ to Trp in Oct-TriA_5_, changes the bioactivity profile from Gram-negative to broad-spectrum activity. Through the screening of analogues substituted at position 9, we subsequently identified the highly potent tridecaptin Oct-His9 that exhibits effective activity against *P. aeruginosa* and at the same time possesses reduced hemolytic and cytotoxic properties. The observed decrease in cytotoxicity and the promising antibacterial properties provide strong grounds for further investigation and development of these analogues as alternative antibiotics for combating infectious diseases associated with antibiotic-resistant bacterial pathogens.

## Methods

### Global Genome Mining of NRPSs

Genomes of all *Paenibacillus* spp. available from RefSeq (release 213)^51^ were downloaded from the NCBI FTP site. All genomes with >400 contigs were considered as low-quality assemblies and were removed from the collection. All genomes were analyzed using AntiSMASH (version 6.0.1)^44^ to obtain BGC predictions. These predictions were then used as input into BiG-SCAPE (version 1.1.4),^46^ for the creation of a sequence similarity network, with distance matrix cutoff set to 0.25. The resulting full network was visualized by Cytoscape (3.9.1).^52^ To predict the specificity of the adenylation domains of NRPS BGCs in this study, we used the software package PARAS (v0.0.4, available at https://github.com/BTheDragonMaster/paras). PARAS is an adenylation domain predictor that uses structure-guided sequence alignments to extract the active site prior to a prediction.

### Genome Sequencing, Assembly, and Annotation of *Paenibacillus* sp. JJ-21

*Paenibacillus* sp. JJ-21 was grown in tryptic soy broth (TSB) at 30 °C and 220 rpm for 24 h. DNA was extracted from the *Paenibacillus* sp. JJ-21 as described.^53^ DNA quality was verified by agarose gel electrophoresis. PacBio sequencing and assembly was performed by Novogene (UK). Generally, libraries were prepared using the SMRTbell template prep kit (PacBio) according to manufacturer instructions. Sequencing was then performed using the PacBio Sequel platform in continuous long reads mode. Assembly was done using Falcon (version 1.8.1).^43^ BGCs in this genome were annotated using AntiSMASH (version 6.0.1).^44^

### Growth of *Paenibacillus* sp. JJ-21 and Secondary Metabolites Extraction

*Paenibacillus* sp. JJ-21 was obtained from the Auburn University Plant-Associated Microbial strain collection and had previously been isolated from the root surface of a field-grown corn plant (*Zea mays*) grown in Dunbar, Nebraska, and maintained as a viable cryostock in a −80 °C freezer. *Paenibacillus* sp. JJ-21 was grown at 30 °C on tryptic soy agar (TSA) for 72 h, and 2 to 3 colonies were inoculated into TSB and incubated at 30 °C overnight. This inoculum (1%) was transferred to 1 L Erlenmeyer flasks containing 400 mL of sterile TSB and fermented at 28 °C while being shaken at 220 rpm for 96 h. Cells were collected by centrifugation (5000*g*, 30 min, 4 °C) and extracted for 6 h with 70% isopropyl alcohol (IPA) supplemented with 0.1% (v/v) formic acid (FA). The crude extracts were clarified by centrifugation, and then the solvent was evaporated under reduced pressure and reconstituted in 50% methanol.

### LC–MS/MS Analysis

For LC–MS analyses, extracts were dissolved in 50% MeOH to a final concentration of 2 mg/mL, and 1 μL was injected into the Shimadzu Nexera X2 UHPLC system coupled to a Shimadzu 9030 QTOF mass spectrometer, and data acquisition was performed as previously described.^54^ Briefly, all of the samples were analyzed in positive polarity, using data-dependent acquisition mode. In this regard, full scan MS spectra (*m*/*z* 100–1700, scan rate 10 Hz, ID enabled) were followed by 2 data-dependent MS/MS spectra (*m*/*z* 100–1700, scan rate 10 Hz, ID disabled) for the 2 most intense ions per scan. The ions were fragmented using collision-induced dissociation (CID) with fixed collision energy (CE 20 eV) and excluded for 1 s before being reselected for fragmentation. The parameters used for the ESI source were an interface voltage of 4 kV, an interface temperature of 300 °C, a nebulizing gas flow of 3 L/min, and a drying gas flow of 10 L/min.

### General Procedure for Manual Solid-Phase Peptide Synthesis

The peptides were made on a 0.25 mmol scale on either preloaded Wang resin or 2-chlorotrityl chloride (CTC) resin. The syntheses of Oct-TriA_1_ and Oct-TriA_5_ were performed on preloaded Fmoc-Ala-Wang resin (0.29 mmol/g loading). The synthesis of Oct-TriD was performed by loading Fmoc-Dab(Boc)–OH on a CTC resin. Resin loading was determined to be 0.50 mmol/g. All couplings with the exception of Fmoc-D-*allo*-Ile-OH and Fmoc-d-Ile-OH were performed using 4 eq. of amino acid or fatty acid, benzotriazol-1-yloxytris(dimethylamino)phosphonium hexafluorophosphate (BOP) (4 equiv), and N,N-diisopropylethylamine (DiPEA) (8 equiv) in 10 mL of DMF for 1 h at RT, under nitrogen flow. Fmoc-D-*allo*-Ile and Fmoc-d-Ile-OH were coupled by treating the resin with 2 equiv of the amino acid, 2 equiv of BOP, and 4 equiv of DiPEA in 10 mL of DMF overnight at RT. Fmoc group removal was performed by treating the resin with 10 mL of piperidine:DMF (1:4, v/v) for 5 min and then again for 15 min. Final side chain deprotection and cleavage from the resin was carried out by treating the resin with 10 mL of TFA:TIPS:H_2_O (95:2.5:2.5, v/v) for 90 min. The reaction mixture was filtered through cotton, and the filtrate was precipitated in MTBE: petroleum ether (1:1, v/v) and centrifuged (4500 rpm, 5 min). The pellet was then resuspended in MTBE: petroleum ether (1:1, v/v) and centrifuged again (4500 rpm, 5 min). Finally, the pellet containing the crude lipopeptide was dissolved in tBuOH:H_2_O (1:1, v/v) and lyophilized overnight. The crude mixtures were subsequently purified by RP-HPLC (for details, see Supporting Information, Methods). Fractions were assessed by HPLC and LC-MS, and the product containing fractions were pooled, frozen, and lyophilized to yield pure lipopeptides in >95% purity (determined by HPLC).

### General Procedure for Automated Solid-Phase Peptide Synthesis

Position 9 analogues were made on a 0.05 mmol scale on preloaded Fmoc-Ala-CTC resin (0.68 mmol/g) using a CEM Liberty Blue automated peptide synthesizer with microwave irradiation. Couplings were performed at 0.125 M concentration using 5 equiv of amino acid, 5 equiv of 1-[bis(dimethylamino)methylene]-1H-1,2,3-triazolo[4,5-*b*]pyridinium 3-oxide hexafluorophosphate (HATU), and 10 equiv of diisopropylethylamine (DIPEA). Fmoc group removal was performed using piperidine:DMF (1:4, v/v). Cleavage from resin along with global deprotection and subsequent purification by RP-HPLC was performed as described above for the manually synthesized peptides.

### Antimicrobial Testing

All minimum inhibitory concentrations (MICs) were determined according to the Clinical and Standards Laboratory Institute (CLSI) guidelines. In brief, agar plates were inoculated from glycerol stocks and incubated overnight at 35 °C. Peptide DMSO stocks were dispensed into 96-well round-bottom plates using a TECAN D300e dispenser. Colistin sulfate (Sigma-Aldrich) was dissolved in water and serially diluted in a cation-adjusted Mueller Hinton broth (10 μL per well). Final concentrations of the test compounds were 0.06 to 64 μg/mL. Inocula were prepared by direct colony suspension in NaCl at 0.5 MacFarland, which was further diluted 200-fold in cation-adjusted Mueller Hinton broth for a target inoculum of 2 × 10^5^ colony forming units (CFUs)/mL. 100 μL portion of the prepared inoculum was added to each well of the prepared plates containing the test compounds. Plates were sealed with parafilm and incubated for 20 h at 35 °C. Images of all plates were recorded, and MIC was assessed visually.

### Hemolysis Assays

Peptides, dissolved in DMSO, were dispensed into 96-well round-bottom microplates using a TECAN D300e dispenser and subsequently diluted with 100 μL of PBS. PBS or 2% Triton X-100 was used as negative and positive controls, respectively. Rabbit red blood cells (BioConcept) were diluted in PBS to a final concentration of 2%, and 100 μL was added to each well of the prepared 96-well plates. The final concentration of the test compounds was 0.25 to 128 μg/mL. Plates were incubated at 37 °C for 1 h and afterward subjected to centrifugation at 1000*g* for 5 min. Then, 30 μL of supernatant was transferred to a new round-bottom plate, and absorbance was measured at 405 nm (TECAN Infinite F200). EC50s were calculated after blank subtraction and normalization to the Triton control.

### Cytotoxicity Determination

HepG2 cells were grown in EMEM (Sigma) supplemented with 2 mM glutamine and 10% fetal bovine serum (Fisher) at 37 °C, 5% CO_2_. 20′000 cells per well were seeded into clear tissue culture-treated 96-well plates and incubated for 24 h. Peptides, dissolved in DMSO, were dispensed into 96 deep well plates (TECAN D300e dispenser) and diluted to a final concentration of 1 to 128 μg/mL using fresh medium without serum. The next day, the old media were removed from the cells, and 200 μL of the prepared compound dilutions was added per well. Plates were incubated for another 24 h before assessing the cell viability using the CellTiter-Glo kit (Promega) according to the manufacturer’s protocol. IC50s were calculated after normalization to the untreated control.

### LIVE/DEAD Staining and Confocal Microscopy

This assay was performed according to a previously described procedure.^55,56^ Briefly, overnight cultures of *E. coli* ATCC 25922 and *S. aureus* ATCC 29213 were diluted to an OD_600_ of 0.2 in MHB and mixed with 1 × MIC of Oct-TriA_1_, Oct-TriA_5_, and Oct-TriD. Nisin and polymyxin B were used as positive controls at a concentration of 2-fold MIC. At the same time, green fluorescent nucleic acid stain SYTO 9 (excitation, 450–490 nm; emission, 500–550 nm) and red fluorescent nucleic acid stain propidium iodide (excitation, 574–599 nm; emission, 612–682 nm) (LIVE/DEAD Baclight Bacterial Viability Kit, Invitrogen) were added to the above cell suspensions. After mixing and incubating for 15 min in the dark at RT, cells were briefly sedimented via centrifugation and resuspended in fresh MHB. Then, the cell suspensions were loaded on 1.5% agarose pads and analyzed by a Zeiss Imager M2 microscope. Confocal images were obtained at 4 random locations for each sample and visualized using FIJI version 1.51H.^57^

## Supporting Information Available

Bioinformatic analyses, HRMS and MS/MS data, protocols and HPLC traces for synthesized peptides, and lipid II antagonization assays (PDF)
