# Supplementary material for: Discovery and Derivatization of Tridecaptin Antibiotics with Altered Host Specificity and Enhanced Bioactivity
Source: ACS Chem Biol. 2024 Apr 11;19(5):1106–15. doi: 10.1021/acschembio.4c00034 (PMC11106739; doi:10.1021/acschembio.4c00034)
Supplement: Supplementary file 1 — cb4c00034_si_001.pdf [file cb4c00034_si_001.pdf]

Supplemental Information

Belonging to the manuscript

Discovery and Derivatization of Tridecaptin Antibiotics with Altered Host Specificity and Enhanced Bioactivity

Nataliia V. Machushynets [a], Karol Al Ayed [b], Barbara R. Terlouw [c], Chao Du [a], Ned P. Buijs [b], Joost Willemse [a], Somayah S. Elsayed [a], Julian Schill [d], Vincent Trebosc [d], Michel Pieren [d], Francesca M. Alexander [e], Stephen A. Cochrane [e], Mark R. Liles [f], Marnix H. Medema [c], Nathaniel I. Martin [b] and Gilles P. van Wezel [a,g,*]

[a] Molecular Biotechnology, Institute of Biology, Leiden University, Leiden 2333 BE, The Netherlands

[b] Biological Chemistry Group, Institute of Biology, Leiden University, Leiden 2333 BE, The Netherlands

[c] Bioinformatics Group, Wageningen University, Wageningen 6708 PB, The Netherlands

[d] BioVersys AG, c/o Technologiepark, Basel CH-4057, Switzerland

[e] School of Chemistry and Chemical Engineering, Queen's University of Belfast, Belfast BT9 5AG, UK

[f] Department of Biological Sciences, Auburn University, Auburn AL 36849, AL, USA

[g] Department of Microbial Ecology, Netherlands Institute of Ecology, Wageningen 6708 PB, The Netherlands

*Author for correspondence. Tel: +31 71 5274310; email: g.wezel@biology.leidenuniv.nl

METHODS

Peptide synthesis

Fmoc-L-Dab(Boc)-OH, Fmoc-D-Dab(Boc)-OH, Fmoc-D-Ile-OH, Fmoc-allo-Ile-OH and 1-[Bis(dimethylamino)methylene]-1H-1,2,3-triazolo[4,5-b]pyridinium 3-oxide hexafluorophosphate (HATU) were purchased from Combi-Blocks. All other Fmoc-amino acids, the Fmoc-Ala-Wang resin and Fmoc-Glu(OtBu)-Wang resin were purchased from P3 BioSystems. 2-Chlorotrityl chloride resin (CTC) was purchased from Iris Biotech. Octanoic acid was purchased from Alfa Aesar. ((1H-Benzo[d][1,2,3]triazol-1-yl)oxy)tris(dimethylamino)phosphonium hexafluorophosphate (BOP), N,N-Diisopropylcarbodiimide (DIC) and triisopropylsilane (TIPS) were purchased from Manchester Organics. Diisopropylethylamine (DIPEA), piperidine, trifluoroacetic acid (TFA) and dimethyl sulfoxide (DMSO) were purchased from Carl Roth. Dichloromethane (CH_2Cl_2) and petroleum ether were purchased from VWR Chemicals. Acetonitrile (MeCN), dimethylformamide (DMF) and methyl tertiary-butyl ether (MTBE) were purchased from Biosolve.

Resin swelling

The resin was swollen in 10 ml of DMF for 300 s prior to the first coupling.

Automated coupling protocol

Step	Function	Duration/Temperature
1	Initial Deprotection N-terminus	15 s at 60°C then 30 s at 70°C
2	Deprotection N-terminus	15 s at 60°C then 180 s at 70°C
3	Wash (DMF)	RT
4	Wash (DMF)	RT
5	Wash (DMF)	RT
6	Coupling amino acid	15 s at 60°C then 300 s at 70°C

After coupling of the final residue on the synthesizer, the resin was washed with DCM, filtered and treated with 3 mL of TFA : TIPS : H_2O (95 : 2.5 : 2.5, v/v) for 90 min. The reaction mixture was filtered through cotton, the filtrate was precipitated in MTBE : petroleum ether (1 : 1, v/v) and centrifuged (4500 rpm, 5 min). The pellet was then resuspended in MTBE : petroleum ether (1 : 1, v/v) and centrifuged again (4500 rpm, 5 min). Finally the pellet containing the crude lipopeptide was dissolved in tBuOH : H_2O (1 : 1, v/v) and lyophilized overnight. The crude mixtures were subsequently purified by RP-HPLC. Fractions were assessed by HPLC and LC-MS and product containing fractions were

pooled, frozen and lyophilized to yield the pure lipopeptides in >95% purity (determined by HPLC).

Prep RP-HPLC purification

Peptides were purified using a BESTA-Technik system with a Dr. Maisch Reprosil Gold 120 C18 column (25 × 250 mm, 10 µm) and equipped with a ECOM Flash UV detector monitoring at 214 nm and 254 nm. The following solvent system, at a flow rate of 12 mL/min, was used: solvent A, 0.1 % TFA in water/acetonitrile 95/5; solvent B, 0.1 % TFA in water/acetonitrile 5/95. Gradient elution was as follows: 100:0 (A/B) for 5 min, 100:0 to 50:50 (A/B) over 50 min, 50:50 to 0:100 (A/B) for 3min, then reversion back to 100:0 (A/B) over 1 min, 100:0 (A/B) for 5 min.

Analytical RP-HPLC

Shimadzu Prominence-i LC-2030 system with a Dr. Maisch ReproSil Gold 120 C18 column (4.6 × 250 mm, 5 µm) at 30 °C and equipped with a UV detector monitoring 214 nm and 254 nm. The following solvent system, at a flow rate of 1 mL/min, was used: solvent A, 0.1 % TFA in water/acetonitrile 95/5; solvent B, 0.1 % TFA in water/acetonitrile 5/95. **Method A**: Gradient elution was as follows: 100:0 (A/B) for 2 min, 100:0 to 0:100 (A/B) over 23 min, 0:100 (A/B) for 1 min, 0:100 (A/B) then reversion back to 100:0 (A/B) over 1 min, 100:0 (A/B) for 3 min. **Method B**: Gradient elution was as follows: 100:0 (A/B) for 2 min, 100:0 to 50:50 (A/B) over 45 min, 50:50 (A/B) to 0:100 (A/B) over 1 min, 0:100 (A/B) for 6 min then reversion back to 100:0 (A/B) over 1min, 100:0 (A/B) for 5min. **Method C:** Gradient elution was as follows: 100:0 (A/B) for 2 min, 100:0 to 0:100 (A/B) over 53 min, 0:100 (A/B) for 1 min, 0:100 (A/B) then reversion back to 100:0 (A/B) over 1 min, 100:0 (A/B) for 4 min.

HRMS analysis

HRMS analyses were performed on a Thermo Scientific Dionex UltiMate 3000 HPLC system with a Phenomenex Kinetex C18 (2.1 x 150 mm, 2.6 µm) column at 35 °C and equipped with a diode array detector. The following solvent system, at a flow rate of 0.3 mL/min, was used: solvent A, 0.1 % formic acid in water; solvent B, 0.1% formic acid in acetonitrile. Gradient elution was as follows: 95:5 (A/B) for 1 min, 95:5 to 5:95 (A/B) over 9 min, 5:95 to 2:98 (A/B) over 1 min, 2:98 (A/B) for 1 min, then reversion back to 95:5 (A/B) over 2 min, 95:5 (A/B) for 1 min. This system was connected to a Bruker micrOTOF-Q II mass spectrometer (ESI ionisation) calibrated internally with sodium formate.

Lipid II antagonization assay

The minimum inhibitory concentrations (MICs) of Oct-TriA$_1$, Oct-TriA$_5$ and vancomycin against *Staphylococcus aureus* USA300 (MRSA) were determined according to Clinical and Standards

Laboratory Institute (CLSI) guidelines. Gram-positive lipid II, containing lysine at position 3 of the pentapeptide, was prepared by total chemical synthesis.[1,2] Lipid II in 1 : 1 chloroform : methanol (v/v) was added to a polypropylene 96-well plate (5-fold molar excess compared to test antibiotics) and the organic solvent was allowed to evaporate. Oct-TriA$_5$ and vancomycin in MHB (50 µL, 16xMIC) were added to the wells with the 5-fold molar excess of pure Gram-positive lipid II in triplicates and to the control wells without lipid II. Single colony of *S. aureus* USA300 (MRSA) from a fresh blood agar plate was suspended in TSB and grown to an OD_{600} of 0.5. The bacterial culture then was diluted in MHB with 0.002% polysorbate-80 to reach 10^6 CFU/mL and 50 µL were mixed with test compounds to achieve the final concentration of 8xMIC for all tested compounds. The plate was incubated at 37°C for 18 h with constant shaking (600 rpm) and subsequently inspected for visible bacterial growth. To visualize the viability of the indicator strain, resazurin sodium salt solution (Merck, Dorset, UK) was added to the cultures to achieve the final concentration of 0.0015% (w/v) and incubated at RT for 1 h.

Table S1 A-domain specificity analysis of NRPS BGCs from the genome of *Paenibacillus* sp. JJ-21.

Domain	Residues in the binding pocket	Amino acid prediction	Probability
Fusaricidin A1	DFWNIGMVH	Threonine	0.716357143
Fusaricidin A2	DAFWLGCTF	Valine	0.795809524
Fusaricidin A3	DASTLAGVC	Tyrosine	0.78
Fusaricidin A4	DFWNIGMVH	Threonine	0.716357143
Fusaricidin A5	DLTKIGEVG	Asparagine	0.96
Fusaricidin A6	DFPNFCIVY	Alanine	0.77
Polymyxin A1	DVGEISSID	2,4-diaminobutyric acid	1
Polymyxin A2	DFWNIGMVH	Threonine	0.716357143
Polymyxin A3	DVGEISSID	2,4-diaminobutyric acid	1
Polymyxin A4	DVGEISSID	2,4-diaminobutyric acid	0.99
Polymyxin A5	DVGEISSID	2,4-diaminobutyric acid	0.99
Polymyxin A6	DAWIVGAIV	Leucine	0.716357143
Polymyxin A7	DFWNIGMVH	Threonine	0.18
Polymyxin A8	DVGEISAID	2,4-diaminobutyric acid	0.716357143
Polymyxin A9	DVGEISAID	2,4-diaminobutyric acid	0.99
Polymyxin A10	DFWNIGMVH	Threonine	0.99
Tridecaptin A1	DAFWLGGTF	Valine	0.661904762
Tridecaptin A2	DVGEISSID	2,4-diaminobutyric acid	0.99
Tridecaptin A3	DILQMGMVW	Glycine	0.98
Tridecaptin A4	DVWHFSLVD	Serine	0.99
Tridecaptin A5	DAWAFAGVA	Tryptophan	0.63
Tridecaptin A6	DVWHFSLVD	Serine	0.99
Tridecaptin A7	DVGEISSID	2,4-diaminobutyric acid	0.99
Tridecaptin A8	DVGEISSID	2,4-diaminobutyric acid	0.99
Tridecaptin A9	DAWAFAGVA	Tryptophan	0.63
Tridecaptin A10	DAKDLGVVD	Glutamic acid	0.66
Tridecaptin A11	DAFWLGGTF	Valine	0.621571429
Tridecaptin A12	DAFFLGITF	Isoleucine	0.815
Tridecaptin A13	DVFWLGGTF	Alanine	0.72

Table S2 A-domain specificity comparison of reference tridecaptin A_1 BGC and tridecaptin cluster from the genome of *Paenibacillus* sp. JJ-21.

**Domain**	**Residues in the binding pocket**		**Amino acid prediction**	
	Tridecaptin A_1 reference BGC BGC0000449	Tridecaptin A_5 BGC of *Paenibacillus* sp. JJ-21	Tridecaptin A_1 reference BGC BGC0000449	Tridecaptin A_5 BGC of *Paenibacillus* sp. JJ-21
Tridecaptin A1	DAFWLGGTF	DAFWLGGTF	Valine/Glycine	Valine
Tridecaptin A2	DVGEISSID	DVGEISSID	2,4-diaminobutyric acid	2,4-diaminobutyric acid
Tridecaptin A3	DILQMGMVW	DILQMGMVW	Glycine	Glycine
Tridecaptin A4	DVWHFSLVD	DVWHFSLVD	Serine	Serine
Tridecaptin A5	DAWAFAGVA	DAWAFAGVA	Tryptophan /Phenylalanine	Tryptophan
Tridecaptin A6	DVWHFSLVD	DVWHFSLVD	Serine	Serine
Tridecaptin A7	DVGEISSID	DVGEISSID	2,4-diaminobutyric acid	2,4-diaminobutyric acid
Tridecaptin A8	DVGEISSID	DVGEISSID	2,4-diaminobutyric acid	2,4-diaminobutyric acid
Tridecaptin A9	DAWTFAGVA	DAWAFAGVA	Phenylalanine /Valine/ Isoleucine	Tryptophan
Tridecaptin A10	DAKDLGVVD	DAKDLGVVD	Glutamic acid	Glutamic acid
Tridecaptin A11	DAFWLGGTF	DAFWLGGTF	Valine	Valine
Tridecaptin A12	DAFFLGITF	DAFFLGITF	Isoleucine	Isoleucine
Tridecaptin A13	DVFWLGGTF	DVFWLGGTF	Alanine	Alanine

Table S3 Predicted amino-acid specificities of the adenylation domains present in representative BGCs of tridecaptin gene cluster families (GCFs) in a BiG-SCAPE sequence similarity network.

Accession number	GCF	A1	A2	A3	A4	A5	A6	A7	A8	A9	A10	A11	A12	A13	Compound
NZ_CP092831.1.region009	1	D-Val	D-Dab	Gly	D-Ser	D-Trp	Ser	Dab	D-Dab	Phe	Glu	Val	D-Ile	Ala	Tridecaptin A_1
NZ_CP048793.1.region008	1	D-Val	D-Dab	Gly	D-Ser	D-Trp	Ser	Dab	D-Dab	Phe	Glu	Val	D-Ile	Ala	Tridecaptin A_1
NZ_CP061172.1.region008	1	D-Val	D-Dab	Gly	D-Ser	D-Trp	Ser	Dab	D-Dab	Phe	Glu	Val	D-Ile	Ala	Tridecaptin A_1
NZ_CP073683.1.region012	1	D-Val	D-Dab	Gly	D-Ser	D-Trp	Ser	Dab	D-Dab	Phe	Glu	Val	D-Ile	Ala	Tridecaptin A_1
NZ_CP097778.1.region009	1	D-Val	D-Dab	Gly	D-Ser	D-Trp	Ser	Dab	D-Dab	Phe	Glu	Val	D-Ile	Ala	Tridecaptin A_1
NZ_JTHP01000234.1.region001	1	D-Val	D-Dab	Gly	D-Ser	D-Trp	Ser	Dab	D-Dab	Phe	Glu	Val	D-Ile	Ala	Tridecaptin A_1
NZ_CP023711.1.region001	1	D-Val	D-Dab	Gly	D-Ser	D-Trp	Ser	Dab	D-Dab	Trp	Glu	Val	D-Ile	Ala	Tridecaptin A_5
NZ_CP025957.1.region010	1	D-Val	D-Dab	Gly	D-Ser	D-Trp	Ser	Dab	D-Dab	Trp	NA	Val	D-Ile	Ala	Tridecaptin A_5
NZ_CP084033.1.region008	1	D-Val	D-Dab	Gly	D-Ser	D-Trp	Ser	Dab	D-Dab	Trp	Glu	Val	D-Ile	Ala	Tridecaptin A_5
NZ_CP086373.1.region009	1	D-Val	D-Dab	Gly	D-Ser	D-Trp	Ser	Dab	D-Dab	Trp	Glu	Val	D-Ile	Ala	Tridecaptin A_5
NC_023037.2.region009	1	D-Val	D-Dab	Gly	D-Ser	D-Trp	Ser	Dab	D-Dab	Trp	Glu	Val	D-Ile	Ala	Tridecaptin A_5
NZ_CP011420.1.region010	1	D-Val	D-Dab	Gly	D-Ser	D-Trp	Ser	Dab	D-Dab	Trp	Glu	Val	D-Ile	NA	Tridecaptin A_5
NZ_CP011512.1.region010	1	D-Val	D-Dab	Gly	D-Ser	D-Trp	Ser	Dab	D-Dab	Trp	Glu	Val	D-Ile	Ala	Tridecaptin A_5
NZ_CP015423.1.region015	1	D-Val	D-Dab	Gly	D-Ser	D-Trp	Ser	Dab	D-Dab	Trp	Glu	Val	D-Ile	Ala	Tridecaptin A_5
NZ_CP017968.3.region011	1	D-Val	D-Dab	Gly	D-Ser	D-Trp	Ser	Dab	D-Dab	Trp	Glu	Val	D-Ile	Ala	Tridecaptin A_5
NZ_CP073682.1.region011	1	D-Val	D-Dab	Gly	D-Ser	D-Trp	Ser	Dab	D-Dab	Trp	Glu	Val	D-Ile	Ala	Tridecaptin A_5
NZ_CP097767.1.region001	1	D-Val	D-Dab	Gly	D-Ser	D-Trp	Ser	Dab	D-Dab	Trp	Glu	Val	D-Ile	Ala	Tridecaptin A_5
NZ_FOYG01000003.1.region001	1	D-Val	D-Dab	Gly	D-Ser	D-Trp	Ser	Dab	D-Dab	Trp	Glu	Val	D-Ile	Ala	Tridecaptin A_5
NZ_JAFIBS010000001.1.region012	1	D-Val	D-Dab	Gly	D-Ser	D-Trp	Ser	Dab	D-Dab	Trp	Glu	Val	D-Ile	Ala	Tridecaptin A_5
NZ_JMLR01000007.1.region003	1	D-Val	D-Dab	Gly	D-Ser	D-Trp	Ser	Dab	D-Dab	Trp	Glu	Val	D-Ile	Ala	Tridecaptin A_5
NZ_NOLA01000018.1.region001	1	D-Val	D-Dab	Gly	D-Ser	D-Trp	Ser	Dab	D-Dab	Trp	Glu	Val	D-Ile	Ala	Tridecaptin A_5
NZ_OXKC02000014.1.region001	1	D-Val	D-Dab	Gly	D-Ser	D-Trp	Ser	Dab	D-Dab	Trp	Glu	Val	D-Ile	Ala	Tridecaptin A_5
NZ_POVS01000007.1.region001	1	D-Val	D-Dab	Gly	D-Ser	D-Trp	Ser	Dab	D-Dab	Trp	Glu	Val	D-Ile	Ala	Tridecaptin A_5
Paenibacillus_SP._MBT_JJ-21. region011	1	D-Val	D-Dab	Gly	D-Ser	D-Trp	Ser	Dab	D-Dab	Trp	Glu	Val	D-Ile	Ala	Tridecaptin A_5
NZ_AMQU01000029.1.region001	1	D-Val	D-Dab	Gly	D-Ser	D-Trp	Ser	Dab	D-Dab	Trp	Glu	Val	D-Ile	Ala	Tridecaptin A_5
NZ_CP040829.1.region014	1	D-Val	D-Dab	Gly	D-Ser	D-Trp	Ser	Dab	D-Dab	Trp	Glu	Val	D-Ile	Ala	Tridecaptin A_5
NZ_CP049598.1.region019	1	D-Val	D-Dab	Gly	D-Ser	D-Trp	Ser	Dab	D-Dab	Trp	Glu	Val	D-Ile	Ala	Tridecaptin A_5
NZ_CP097766.1.region009	1	D-Val	D-Dab	Gly	D-Ser	D-Trp	Ser	Dab	D-Dab	Trp	Glu	Val	D-Ile	Ala	Tridecaptin A_5
NZ_CP097771.1.region013	1	D-Val	D-Dab	Gly	D-Ser	D-Trp	Ser	Dab	D-Dab	Trp	Glu	Val	D-Ile	Ala	Tridecaptin A_5
NZ_JAACZY010000021.1.region001	1	D-Val	D-Dab	Gly	D-Ser	D-Trp	Ser	Dab	D-Dab	Trp	Glu	Val	D-Ile	Ala	Tridecaptin A_5
NZ_JAAMNS010000007.1.region001	1	D-Val	D-Dab	Gly	D-Ser	D-Trp	Ser	Dab	D-Dab	Trp	Glu	Val	D-Ile	Ala	Tridecaptin A_5
NZ_JNCB01000011.1.region006	1	D-Val	D-Dab	Gly	D-Ser	D-Trp	Ser	Dab	D-Dab	Trp	Glu	Val	D-Ile	Ala	Tridecaptin A_5
NZ_JTHO01000008.1.region001	1	D-Val	D-Dab	Gly	D-Ser	D-Trp	Ser	Dab	D-Dab	Trp	Glu	Val	D-Ile	Ala	Tridecaptin A_5
NZ_JWJJ01000007.1.region001	1	D-Val	D-Dab	Gly	D-Ser	D-Trp	Ser	Dab	D-Dab	Trp	Glu	Val	D-Ile	Ala	Tridecaptin A_5

NZ_QVPU01000010.1.region001	1	D-Val	D-Dab	Gly	D-Ser	D-Trp	Ser	Dab	D-Dab	Trp	Glu	Val	D-Ile	Ala	Tridecaptin A_5
NC_015690.1.region011	2	D-Phe	D-Dab	D-Ile	D-Ser	D-Trp	Ser	Ser	D-Dab	Trp	Ser	Val	D-Ile	Dab	Tridecaptin D
NC_016935.1.region011	2	D-Phe	D-Dab	D-Ile	D-Ser	D-Trp	Ser	Ser	D-Dab	Trp	Ser	Val	D-Ile	Dab	Tridecaptin D
NC_017672.3.region011	2	D-Phe	D-Dab	D-Ile	D-Ser	D-Trp	Ser	Ser	D-Dab	Trp	Ser	Val	D-Ile	Dab	Tridecaptin D
NZ_JAMAVM010000004.1.region001	3	D-Val	D-Dab	D-Ala	D-Dab	D-Trp	Ser	Asp	D-Dab	Trp	Asp	Val	D-Val	Gln	Tridecaptin G

Table S4 HPLC and HRMS analysis of peptides.

Peptide	Name	Chemical Formula	Calcd. Exact Mass	Mass found	Calcd.	Overall Yield [%]
**1**	Oct-TriA$_1$	$C_{72}H_{113}N_{17}O_{19}$	1519,8399	760,9278 $[M+2H]^{2+}$	760,9272	19
**2**	Oct-TriA$_5$	$C_{74}H_{114}N_{18}O_{19}$	1558,8508	780,4332 $[M+2H]^{2+}$	780,4327	23
**3**	Oct-TriD	$C_{80}H_{120}N_{18}O_{19}$	1636,8977	819,4565 $[M+2H]^{2+}$	819,4562	51
**4**	Oct-Gly9	$C_{65}H_{107}N_{17}O_{19}$	1429,7929	715,9046 $[M+2H]^{2+}$	715,9038	10
**5**	Oct-Ala9	$C_{66}H_{109}N_{17}O_{19}$	1443,8086	722,9120 $[M+2H]^{2+}$	722,9116	11
**6**	Oct-Val9	$C_{68}H_{113}N_{17}O_{19}$	1471,8399	736,9278 $[M+2H]^{2+}$	736,9272	11
**7**	Oct-Ile9	$C_{69}H_{115}N_{17}O_{19}$	1485,8555	743,9357 $[M+2H]^{2+}$	743,9351	16
**8**	Oct-Ser9	$C_{66}H_{109}N_{17}O_{20}$	1459,8035	730,9098 $[M+2H]^{2+}$	730,9090	16
**9**	Oct-Tyr9	$C_{72}H_{113}N_{17}O_{20}$	1535,8348	768,9256 $[M+2H]^{2+}$	768,9247	16
**10**	Oct-Glu9	$C_{68}H_{111}N_{17}O_{21}$	1501,8140	751,9150 $[M+2H]^{2+}$	751,9143	12
**11**	Oct-His9	$C_{69}H_{111}N_{19}O_{19}$	1509,8304	755,9232 $[M+2H]^{2+}$	755,9225	16
**12**	Oct-Dab9	$C_{67}H_{112}N_{18}O_{19}$	1472,8351	737,4254 $[M+2H]^{2+}$	737,4249	12

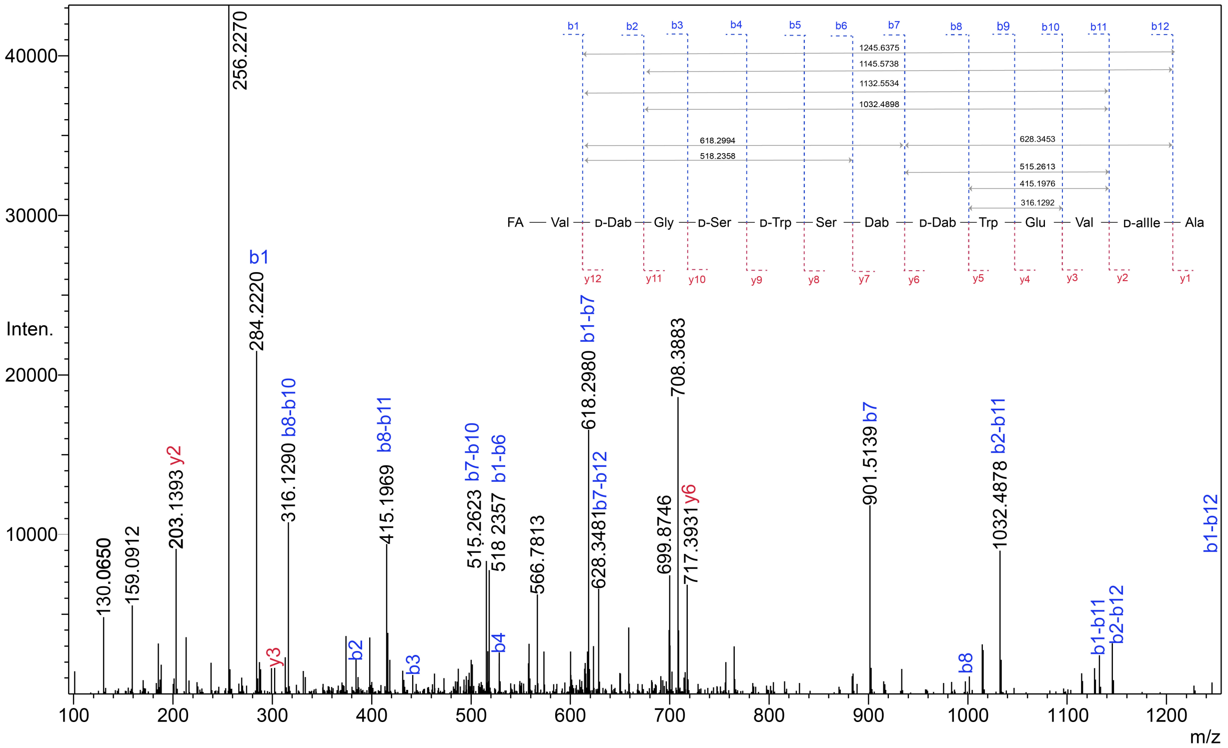

Figure S1. MS/MS spectrum of tridecaptin A$_5$ produced by of *Paenibacillus* sp. JJ-21 (precursor ion $[M+3H]^{3+}$ *m/z* 539.9712). The assignment of the sequence of amino acid residues at the top of the spectrum is based on the mass differences between the consecutive *y* and *b* ions.

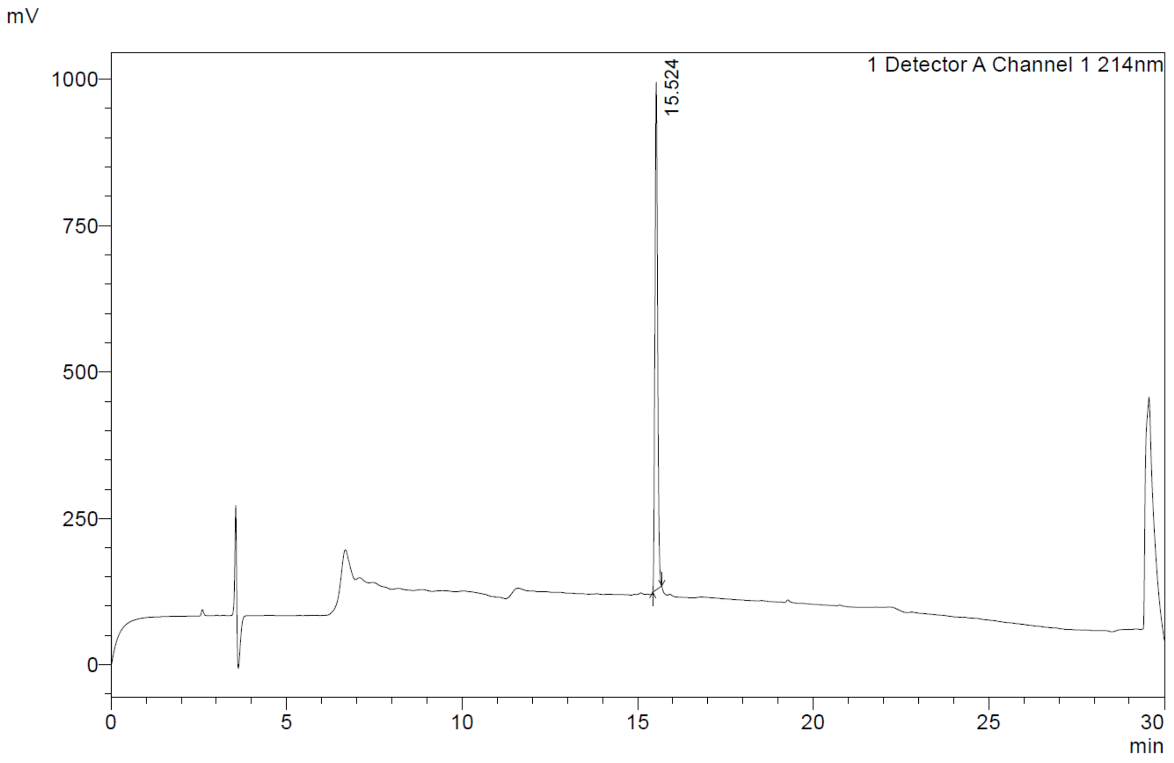

Figure S2. HPLC trace showing the reinjection of purified Oct-TriA$_1$ (**1**). The peptide eluted as a single peak at 15.524 min using the HPLC method A.

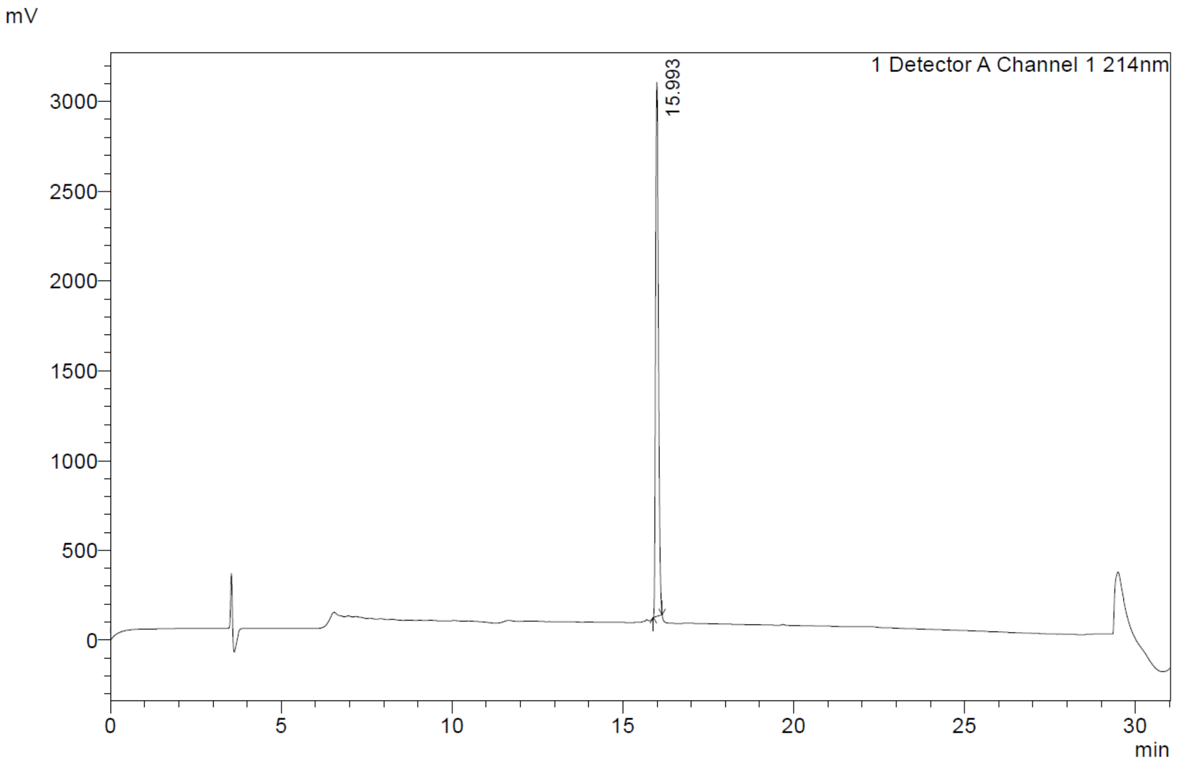

Figure S3. HPLC trace showing the reinjection of purified Oct-TriA$_5$ (**2**). The peptide eluted as a single peak at 15.993 min using the HPLC method A.

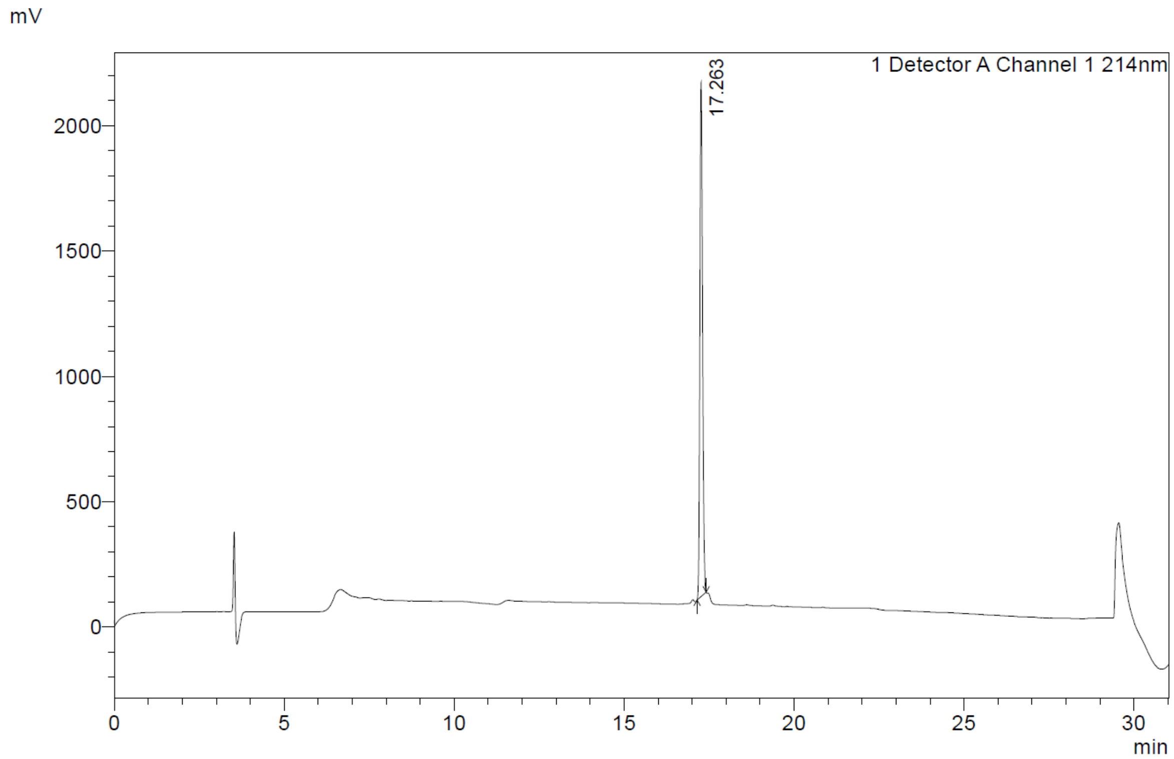

Figure S4. HPLC trace showing the reinjection of purified Oct-TriD (**3**). The peptide eluted as a single peak at 17.263 min using the HPLC method A.

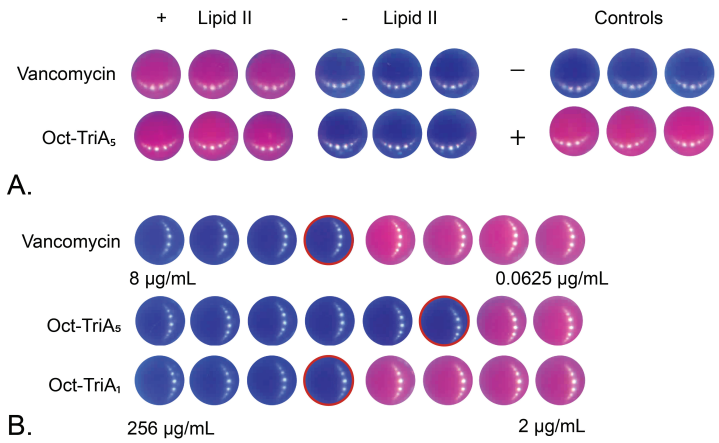

Figure S5. **A.** Gram-positive lipid II binding assays with *S. aureus* USA300 (MRSA) to show lipid II binding of Oct-TriA$_5$. Vancomycin was used as a positive control for lipid II binding. Resazurin was used to visualize the viability of the indicator strain (pink colour indicates growth and blue means inhibition of growth).The addition of lipid II significantly reduced the efficacy of Oct-TriA5 and vancomycin, as the growth of *S. aureus* USA300 (MRSA) was not inhibited at the concentration of 8x MIC. This indicates that Oct-TriA5 binds to Lipid II of Gram-positive bacteria. **B.** MIC assays for Oct-TriA$_1$, Oct-TriA$_5$ and vancomycin against *S. aureus* USA300. The wells with the MIC of the test compounds are highlighted with red edge.

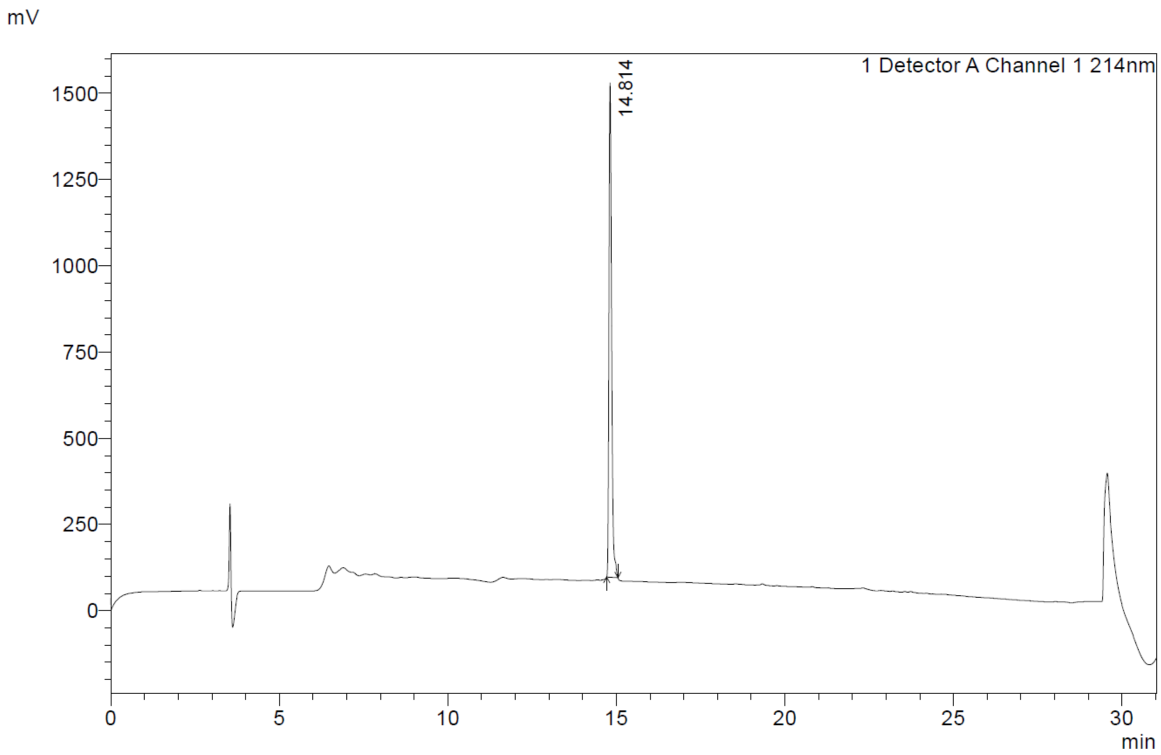

Figure S6. HPLC trace showing the reinjection of purified Oct-Gly9 (**4**). The peptide eluted as a single peak at 14.814 min using the HPLC method A.

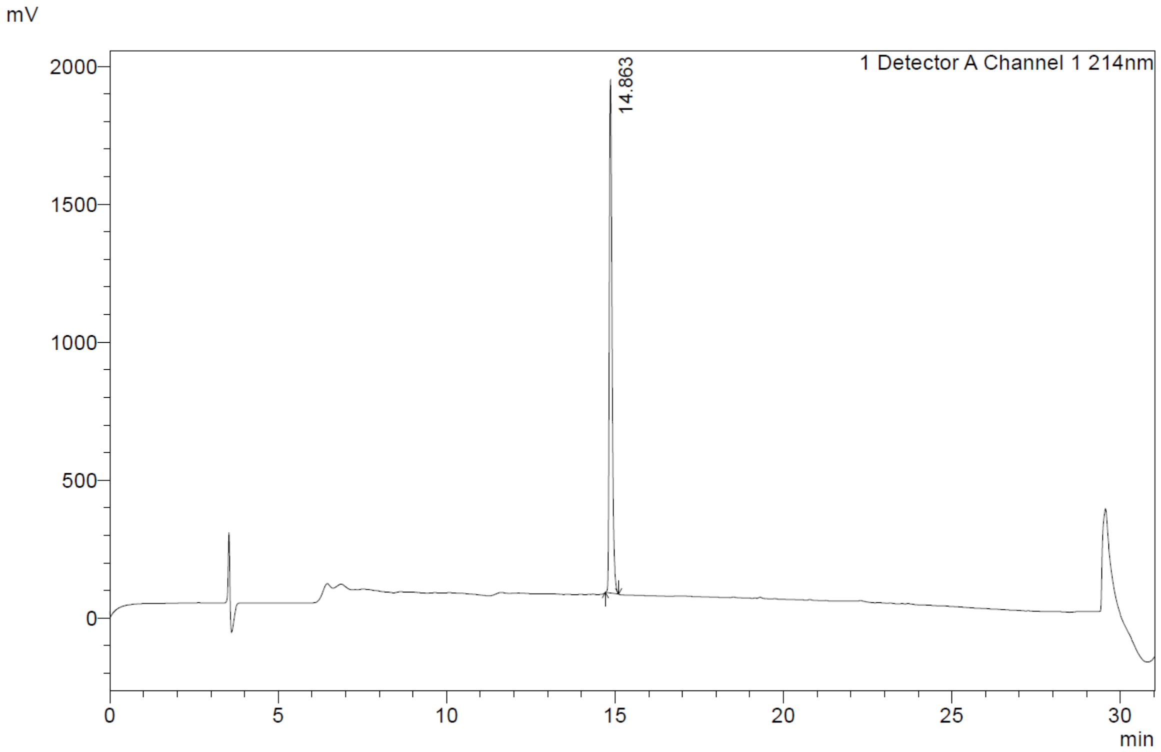

Figure S7. HPLC trace showing the reinjection of purified Oct-Ala9 (**5**). The peptide eluted as a single peak at 14.863 min using the HPLC method A.

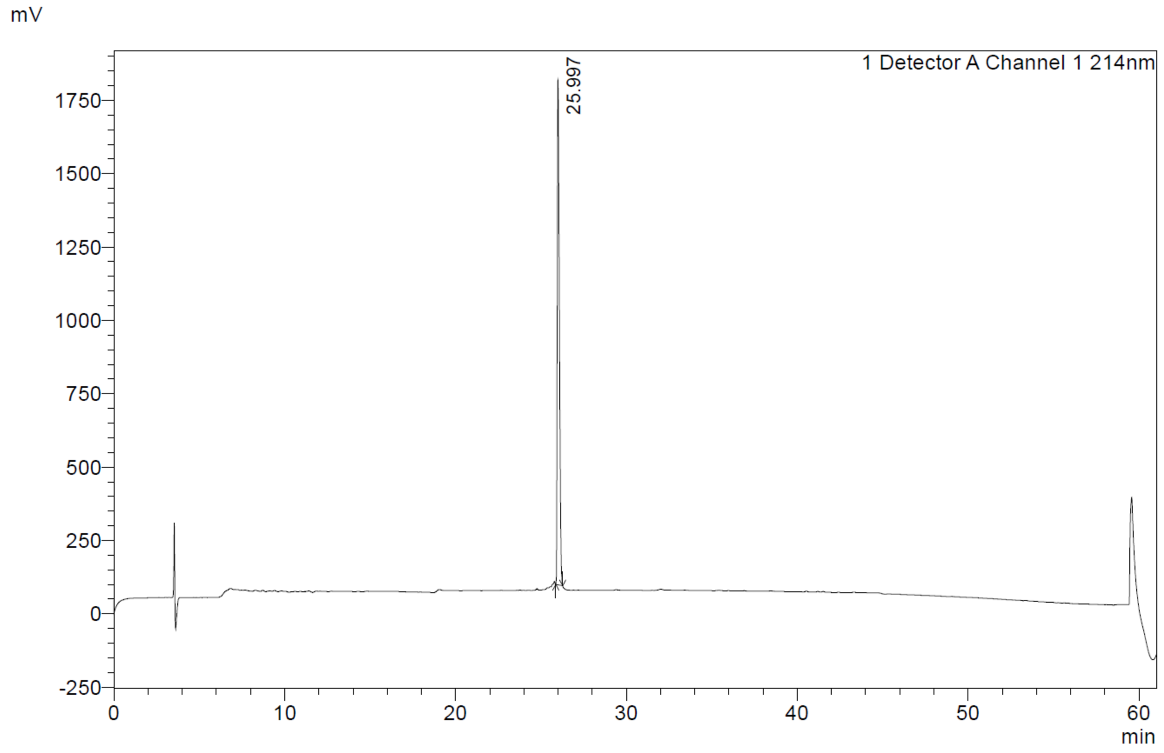

Figure S8. HPLC trace showing the reinjection of purified Oct-Val9 (**6**). The peptide eluted as a single peak at 25.997 min using the HPLC method C.

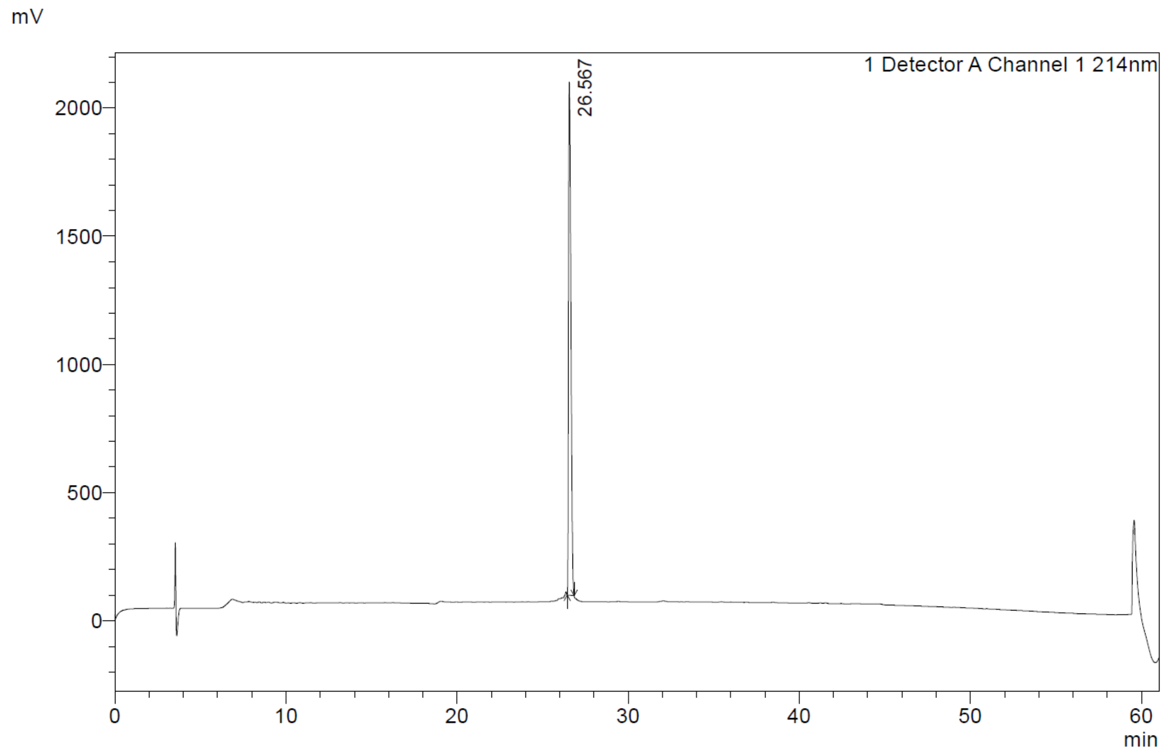

Figure S9. HPLC trace showing the reinjection of purified Oct-Ile9 (**7**). The peptide eluted as a single peak at 26.567 min using the HPLC method C.

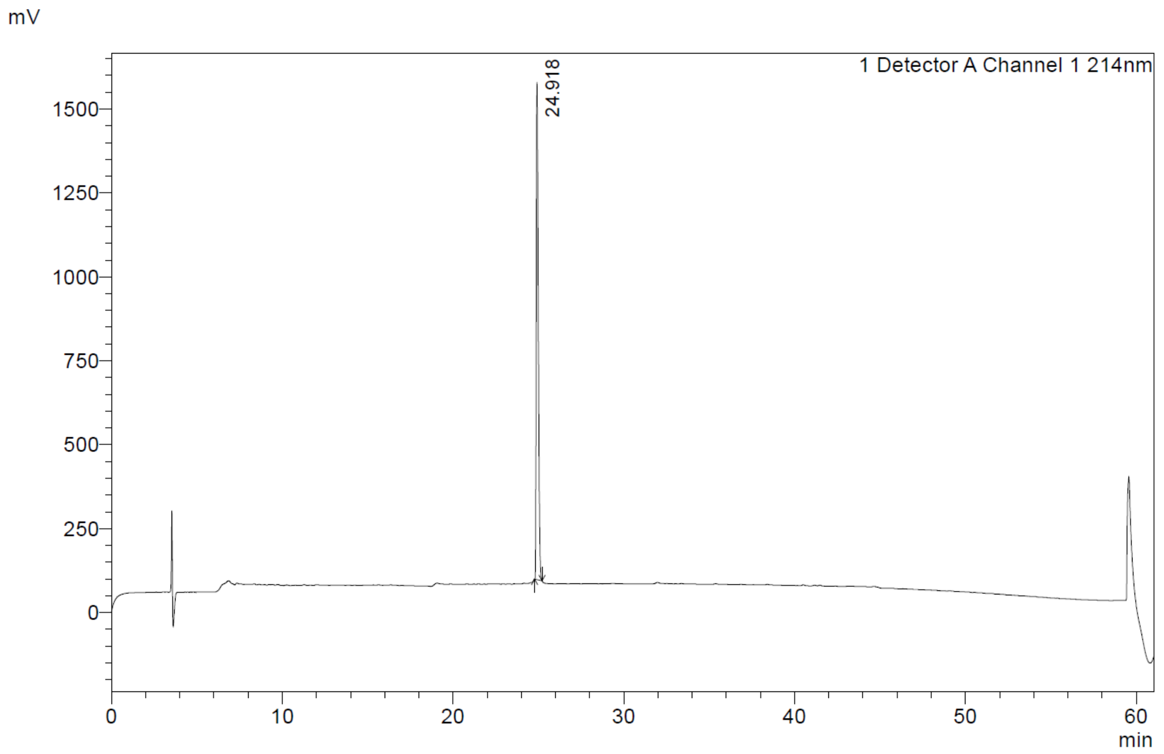

Figure S10. HPLC trace showing the reinjection of purified Oct-Ser9 (**8**). The peptide eluted as a single peak at 24.918 min using the HPLC method C.

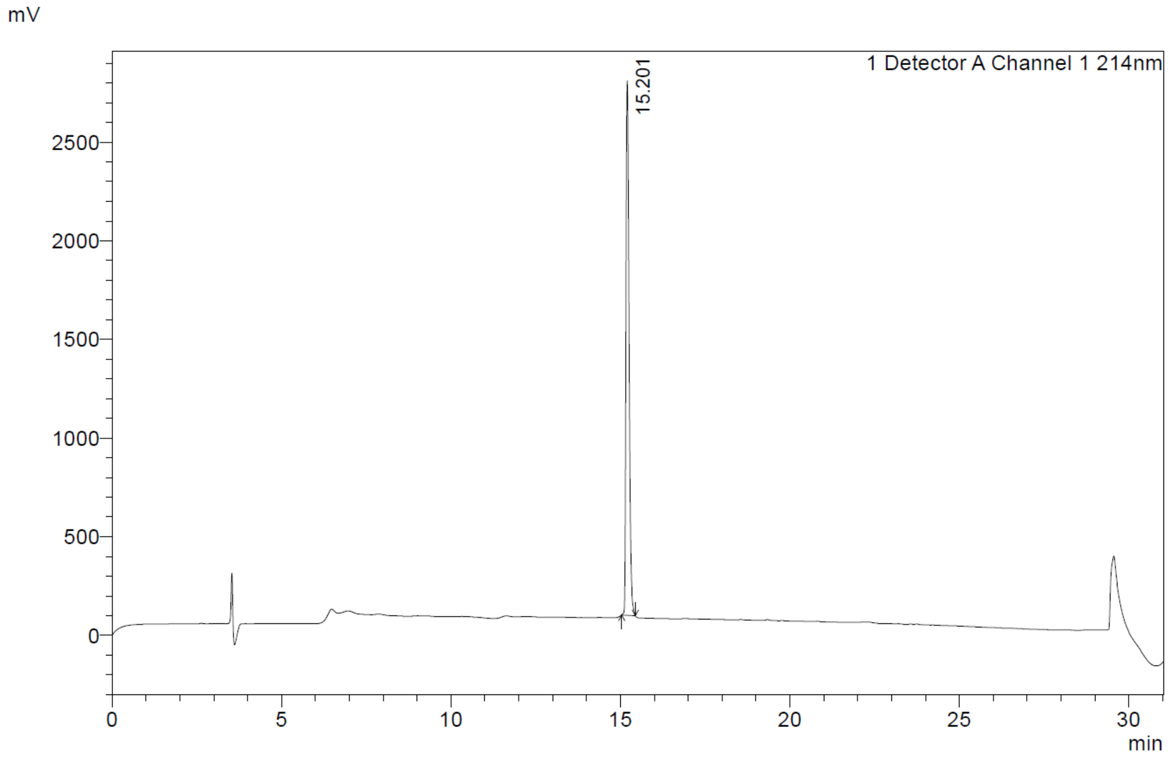

Figure S11. HPLC trace showing the reinjection of purified Oct- Tyr9 (**9**). The peptide eluted as a single peak at 15.201 min using the HPLC method A.

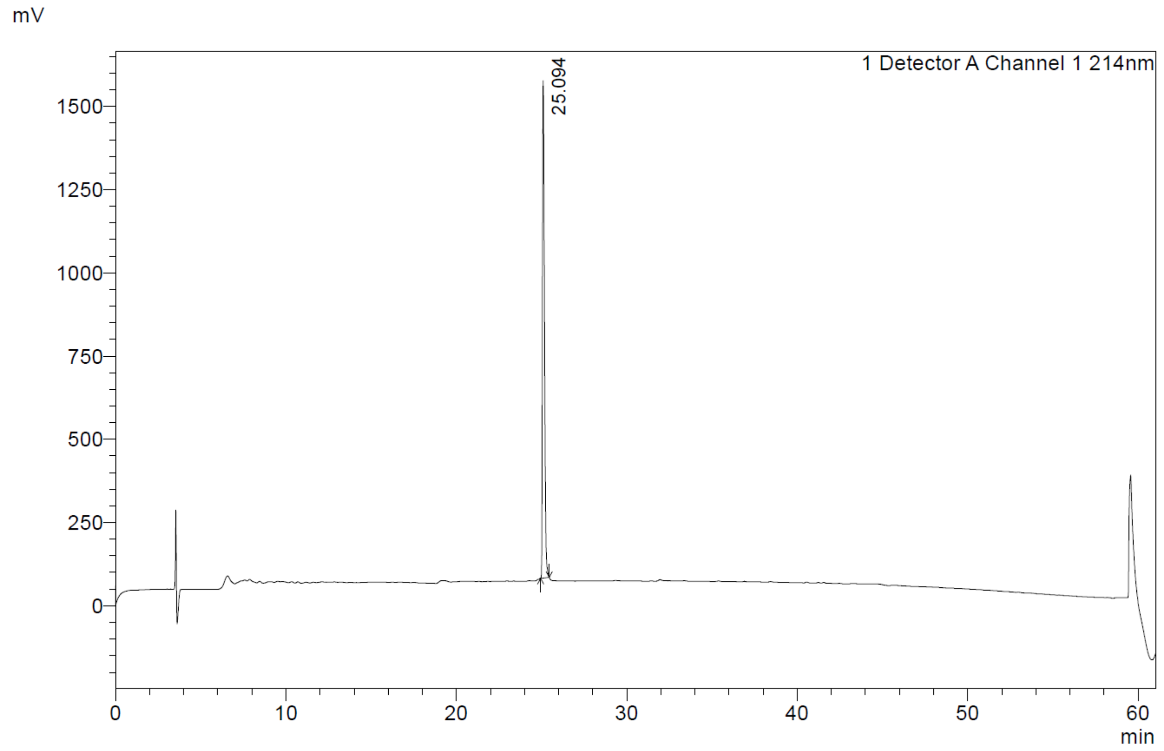

Figure S12. HPLC trace showing the reinjection of purified Oct-Glu9 (**10**). The peptide eluted as a single peak at 25.094 min using the HPLC method C.

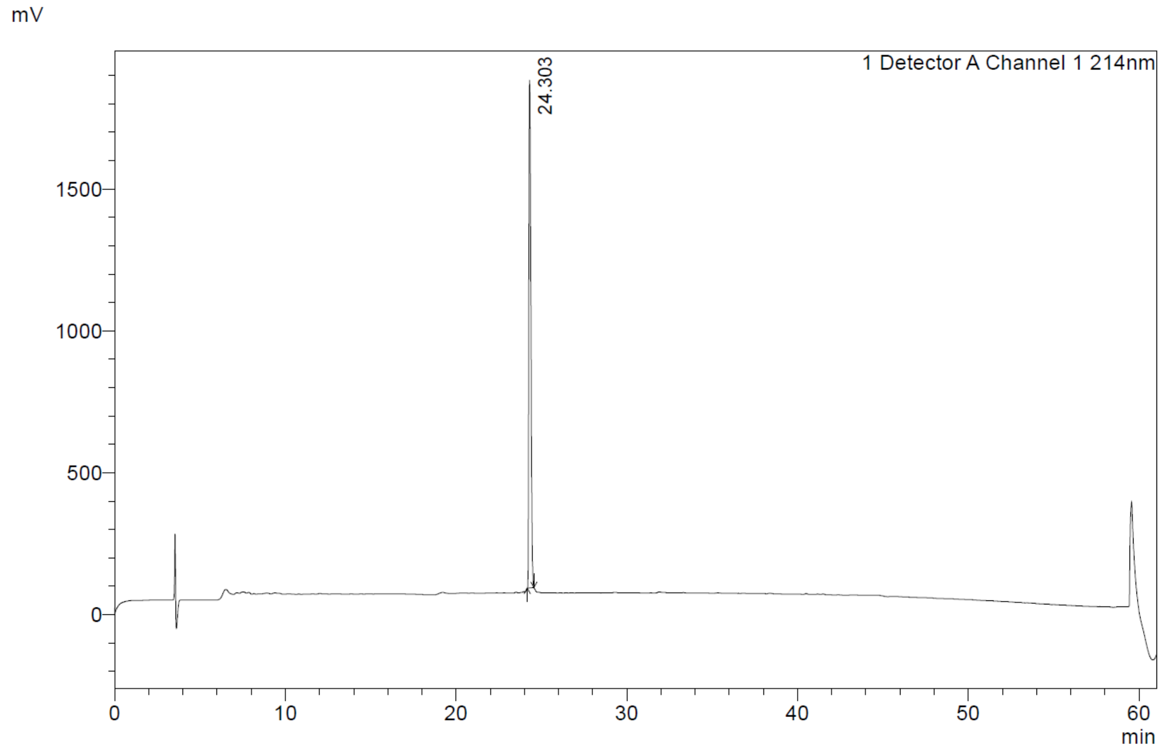

Figure S13. HPLC trace showing the reinjection of purified Oct-His9 (**11**). The peptide eluted as a single peak at 24.303 min using the HPLC method C.

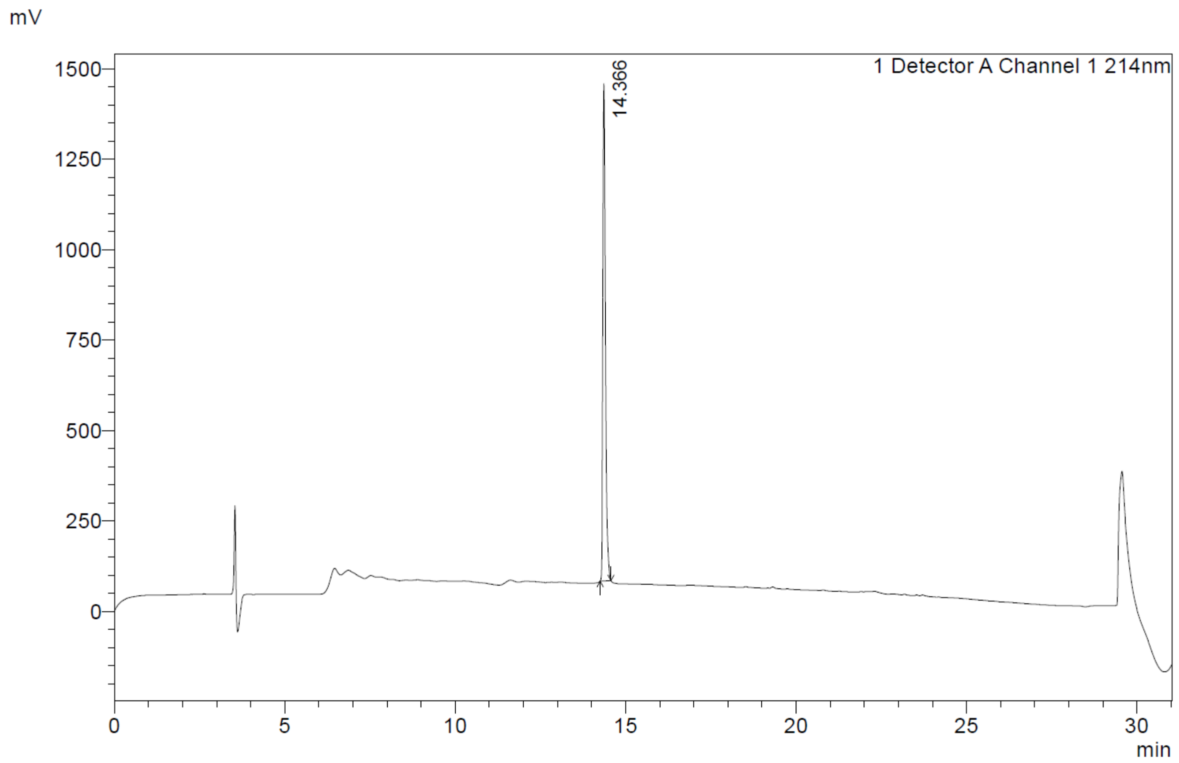

Figure S14. HPLC trace showing the reinjection of purified Oct-Dab9 (**12**). The peptide eluted as a single peak at 14.366 min using the HPLC method A.

REFERENCES

(1) Dong, Y. Y.; Wang, H.; Pike, A. C. W.; Cochrane, S. A.; Hamedzadeh, S.; Wyszyński, F. J.; Bushell, S. R.; Royer, S. F.; Widdick, D. A.; Sajid, A.; et al. Structures of DPAGT1 explain glycosylation disease mechanisms and advance TB antibiotic design. *Cell* **2018**, *175* (4), 1045-1058.e1016. DOI: 10.1016/j.cell.2018.10.037.
(2) Karak, M.; Cloonan, C. R.; Baker, B. R.; Cochrane, R. V. K.; Cochrane, S. A. Optimizations of lipid II synthesis: an essential glycolipid precursor in bacterial cell wall synthesis and a validated antibiotic target. *Beilstein J. Org. Chem* **2024**, *20*, 220-227. DOI: 10.3762/bjoc.20.22.
